# A leakage-aware and interpretable machine learning framework for ovarian cancer prediction using the PLCO screening dataset

**DOI:** 10.3389/fonc.2026.1926361

**Published:** 2026-10-30

**Authors:** Sheela Lavanya J. M., Subbulakshmi P.

**Affiliations:** 1 School of Computer Science and Engineering, Vellore Institute of Technology, Chennai, India; 2 Centre for Advanced Materials and Innovative Technologies, School of Computer Science and Engineering, Vellore Institute of Technology, Chennai, India

**Keywords:** ensemble learning, explainable AI (XAI), feature selection, machine learning, ovarian cancer prediction, stacking classifier

## Abstract

Early diagnosis of ovarian cancer is a significant clinical problem, and this is mainly because there are no strongly sensitive early-detection biomarkers, and the standard screening parameters like CA-125 blood tests and transvaginal ultrasounds are very insensitive in making a diagnosis. This paper presents a machine learning architecture, which is organized and interpretable based on the Prostate, Lung, Colorectal, and Ovarian (PLCO) Cancer Screening data to enhance the risk stratification of ovarian cancer. The original analytical cohort comprised 78,209 participants, including 518 ovarian cancer cases. To select 20 informative pre-diagnostic predictors in a high dimensional feature space, a comprehensive feature selection pipeline with ANOVA (f classifier), Chi-square, Random Forest importance, and XGBoost importance were used. Several classification algorithms were tested and the boosting based methods proved to have a good predictive value. A stacking ensemble of K-Nearest Neighbors and AdaBoost was the highest performing overall (accuracy: 0.7644), and a test-set AUROC of 0.8399, with additional evaluation using sensitivity, specificity, PPV, NPV, balanced accuracy, MCC, and PR-AUC. SHAP analysis and permutation importance were used to provide model interpretability and consistently regarded questionnaire-based screening variables, age, and CA-125 attributes as the strongest ones. The suggested framework offers a methodologically rigorous and transparent method of assessment of population level ovarian cancer risk, providing a foundation for future research toward a clinically useful ovarian cancer screening decision-support system, pending external validation.

## 1 Introduction

Ovarian cancer is one of the most common types of cancers across the globe because it is one of the deadliest cancers of the gynecological type and is mainly attributed by the asymptomatic nature at the early stages and the absence of proper screening procedures in the communities. It has been estimated that the incidence of ovarian cancer is about 300,000 new cases per year and the death rate is over 200,000 annually, which is the eighth most frequent cancer-related death among women in the world (1). The outcome of the disease is closely related to the stage at which the cancer is diagnosed: the overall survival rate of patients diagnosed with the disease at an early stage (Stage I–II) was over 90 percent, and that of patients diagnosed with the disease at an advanced stage was less than 30 percent (2). Due to unclear symptoms and the low level of diagnostic specificity of the available biomarkers and imaging methods, nearly 70 percent of cases are diagnosed at an advanced stage (3).

Current screening methods including transvaginal ultrasound (TVU) and the CA-125 biomarker lack specificity and sensitivity adequacy particularly in asymptomatic women (4). The results of the clinical trials of the PLCO Prostate, Lung, Colorectal, and Ovarian Cancer Screening Trial and the UK Collaborative Trial of Ovarian Cancer Screening (UKCTOCS) showed that CA-125 and transvaginal ultrasound (TVU) were not associated with a statistically significant reduction in mortality in cases of universal screening (5, 6). These limitations necessitate more advanced prediction models that can be used to support early health risk assessment. These models can include different clinical, demographic and biomarker variables.

Machine learning (ML) is a valuable tool for prediction of ovarian cancer due to its ability to learn complex nonlinear relationships in high-dimensional clinical data (7). Logistic regression, support vector machine, Random Forests, gradient boosting, and deep learning models were researched in the past, but these studies were usually limited by a small amount of data, specific biomarkers, or the possible insufficient interpretation of the model (8, 9). The recent studies have identified ensemble learning and advanced feature engineering as a way to improve predictive accuracy (10). However, no comprehensive assessment of extensive, population-based datasets has been performed to date.

As a result of the PLCO dataset, we have the opportunity to fill some of these critical gaps by providing longitudinal screening data, demographic factors, questionnaire responses, and clinical outcomes from over 150,000 people (11). In order to develop more resilient predictive models, a comprehensive dataset should be combined with sophisticated machine learning techniques. Another key element of the XAI methodology is to use explainable artificial intelligence (XAI) techniques like SHAP values and permutation importance to improve transparency and aid potential future clinical application (12, 13).

The aim of this paper is to suggest an integrated system that incorporates multi-method feature selection, machine learning classifiers, ensemble stacking, and the interpretation with XAI to enhance the prediction of ovarian cancer using the PLCO screening dataset. A few key contributions of this paper are:

- Suggested a multi-criteria feature selection method that combines the features selected by ANOVA, Chi-square analysis, features selected by Random Forest importance and features selected by XGBoost importance for selecting stable, non-redundant clinically meaningful features from a high dimensional screening dataset.
- Compared classical machine learning approaches, ensemble methods and boosting algorithms and made the systematic selection of the most effective predictive method of ovarian cancer risk estimation.
- Shown that a stacking ensemble scheme can perform better than single classifiers by attaining better predictive balance and generalization than standalone classifiers and previous benchmarks in the literature.
- Added several interpretability tools including SHAP analysis, permutation importance and cross validation based validation to ensure transparency of the feature relevance and enhance the trustworthiness of the clinical insights from the model.

In this work we provide a comprehensible and evidence-based response to the shortcomings of the existing studies on ovarian cancer screening, that may help to inform the ovarian cancer detection journey.

The rest of this paper is organized as follows. Section 2 provides a summary of the existing literature on the risk prediction of ovarian cancer, machine learning applications for cancer screening, and XAI applications for cancer clinical decision support. The materials and methods are detailed in Section 3, which consists of the PLCO dataset, leakage-aware data preprocessing, multi-criteria feature selection strategy, model development, stacking ensemble framework and evaluation metrics. In Section 4, results are provided for the baseline and post-feature-selection performance, stacking ensemble performance, and interpretability analysis using SHAP and permutation importance. The findings are discussed in the context of current research in Section 5, the methodological contributions, the clinical relevance and the limitations are emphasized. Finally, Section 6 concludes the paper with key insights and outlines directions for future work.

## 2 Related work

This part gives a summary of the recent studies and strategies in the diagnosis and prediction of ovarian cancer. Numerous studies discuss the application of machine learning (ML) models and their understandability, along with the application of biomarkers, such as CA-125, to diagnose cancer at an early stage.

Structured clinical information is one of the first applications to be thought over during the early years of machine learning application in the ovarian cancer prediction and risk stratification. To select features, the authors of the study (14) used 49 clinical variables collected on 349 Chinese patients using the minimal redundancy maximal relevance (MRMR) and decision tree methods. In their results, logistic regression was significantly better than the traditional diagnostic methods, including Risk of Ovarian Malignancy Algorithm (ROMA) and the Risk of Ovarian Cancer Algorithm (ROCA) (15). Owing to the results of this research it is clear that even simple machine learning classifiers can be used as useful decision support systems in case they are properly paired with feature selection.

Kawakami et al. (16) reported the findings of a major study; more than 300 epithelial ovarian cancer cases and over 100 benign tumors were studied, and a variety of machine learning classifiers, such as Support Vector Machines (SVM), Random Forests (RF), Naive Bayes, Elastic Net, and Gradient Boosting, were used. Integrating blood biomarkers into machine learning models led to a substantial improvement in predictive performance as opposed to using traditional regression models. In a similar manner, Paik et al. (17) demonstrated that gradient boosting can be used to categorize prognostic subgroups of EOC patients on the basis of their clinical and pathological characteristics.

Chen (18) has examined the L2-regularized logistic regression model to determine whether women with ovarian cancer in stages I or II are at a higher mortality risk. Through the inclusion of clinical factors into their model, they demonstrated that machine-learning-driven methodologies are capable of providing a higher level of prognostic accuracy than conventional statistical approaches. These results support the contention that machine learning-driven methodologies can strengthen prognostic accuracy. These studies demonstrate that machine learning is capable of diagnosing and assessing risks based on structured medical data, and that it has significant advantages over other methods.

Early detection of cancer can be achieved through the use of blood-derived biomarkers, which are minimally invasive. A laser-induced breakdown spectroscopy (LIBS) and machine learning model classifier analysis was conducted by Yue et al. (19), to evaluate the accuracy of their discriminatory models using blood plasma. As demonstrated by Wibowo et al. (20) in their evaluation of KNN and SVM methodologies in ovarian cancer detection, KNN proved to be more effective than SVM in detecting ovarian cancer from blood characteristics.

As reported by Ma et al. (21), spreading tumor cells (an indicator of health) significantly enhanced the prediction capability of machine learning models such as random forests, support vector machines, gradient boosting, and neural networks. It has been demonstrated that ensemble-based methodologies are more effective than conventional regression techniques when used to solve biological categorization problems.

According to Muinao et al. (22), individual biomarkers, such as CA-125 or HE4, do not possess adequate sensitivity to detect early-stage diseases. It is recommended in their review that multi-marker panels and machine learning-driven integration be developed in order to improve the sensitivity and specificity of diagnostic tests.

Due to the complexity of the genomic data sets, several methods were developed for selecting the most important genes of interest involved in the progression of ovarian cancer (23). In a study by Cho and Won (24), correlation-based metrics, mutual information, and signal-to-noise ratios were evaluated as well as a variety of classifiers, including SVM, MLP, kNN, and SOM, for the ranking of genes. Xu et al. (25) proposed a hybrid approach which integrates particle swarm optimization (PSO) and probabilistic neural networks (PNN) for prediction of survival in a patient with lymphoma. The combination of these two methods led to good classification accuracy in the case of patients with lymphoma.

Sahu and Mishra (26) used a PSO-based gene selection which gave them almost 100% accuracy with SVM and kNN classifiers. Gunavathi et al. (27) compared the swarm intelligence algorithms (SLA), such as Cuckoo search, Shuffled frog leaping, and SFLLF with the best algorithm combination being SFLLF and kNN.

In the same regard, Yasodha and Ananthanarayan (28) employed a hybrid particle-genetic-swarm algorithm in enhancing gene selection in classifying the OC stages with multiclass SVM performing better than ANN and Naive Bayes. These papers emphasize the significance of the application of optimization in feature selection in cases of high-dimensional biomedical data.

Explainability is a requirement in the adoption of machine learning as it approaches more complexities. Kourou et al. (29) applied SHAP and LIME to interpretability to gynecologic cancer data and demonstrated increased trust in feature interpretation with transparency of feature contributions by clinicians. Neural networks based on attention were created by Das et al. (30) and demonstrated biologically relevant gene signatures in relation to OC progression. XAI systems can assist in narrowing the disparity between the performance and clinical usefulness of algorithms, which can guarantee that the predictions offered by ML can be interpreted and held accountable.

### 2.1 Research gap

Even with these improvements, there are a number of drawbacks. Most studies are small, single-center and include only a few biomarkers, or are poorly validated in diverse populations. Moreover, few studies combine multi-method feature selection, ensemble learning and model explainability in a large population scale screening cohort. These limitations must be overcome in the creation of clinically useful and widely applicable ovarian cancer risk prediction models. The present study aims to address these challenges, suggesting a leakage-aware, interpretable machine learning framework with the PLCO screening data. A multi-criteria feature selection technique is a part of this methodology, along with ensemble and stacking models and comprehensive explainability analysis, which enables clinically relevant and strong ovarian cancer risk stratification.

## 3 Methodology

The conceptual framework adopted in the present study comprises of 5 modules, which function synergously and in sequence to gradually transform the raw screening data into a clinically meaningful and accurate ovarian cancer prediction model. **Figure 1** Multi Stacking Framework describes the process of this overall workflow. It begins with the acquisition of the PLCO Screening Dataset, a large population-based cohort that has comprehensive demographic, lifestyle, clinical, biomarker, imaging, screening, and mortality data. This rich data set allows investigation of intricate relationships between multiple data domains that are not commonly found in smaller clinical cohorts.

**Figure 1:**
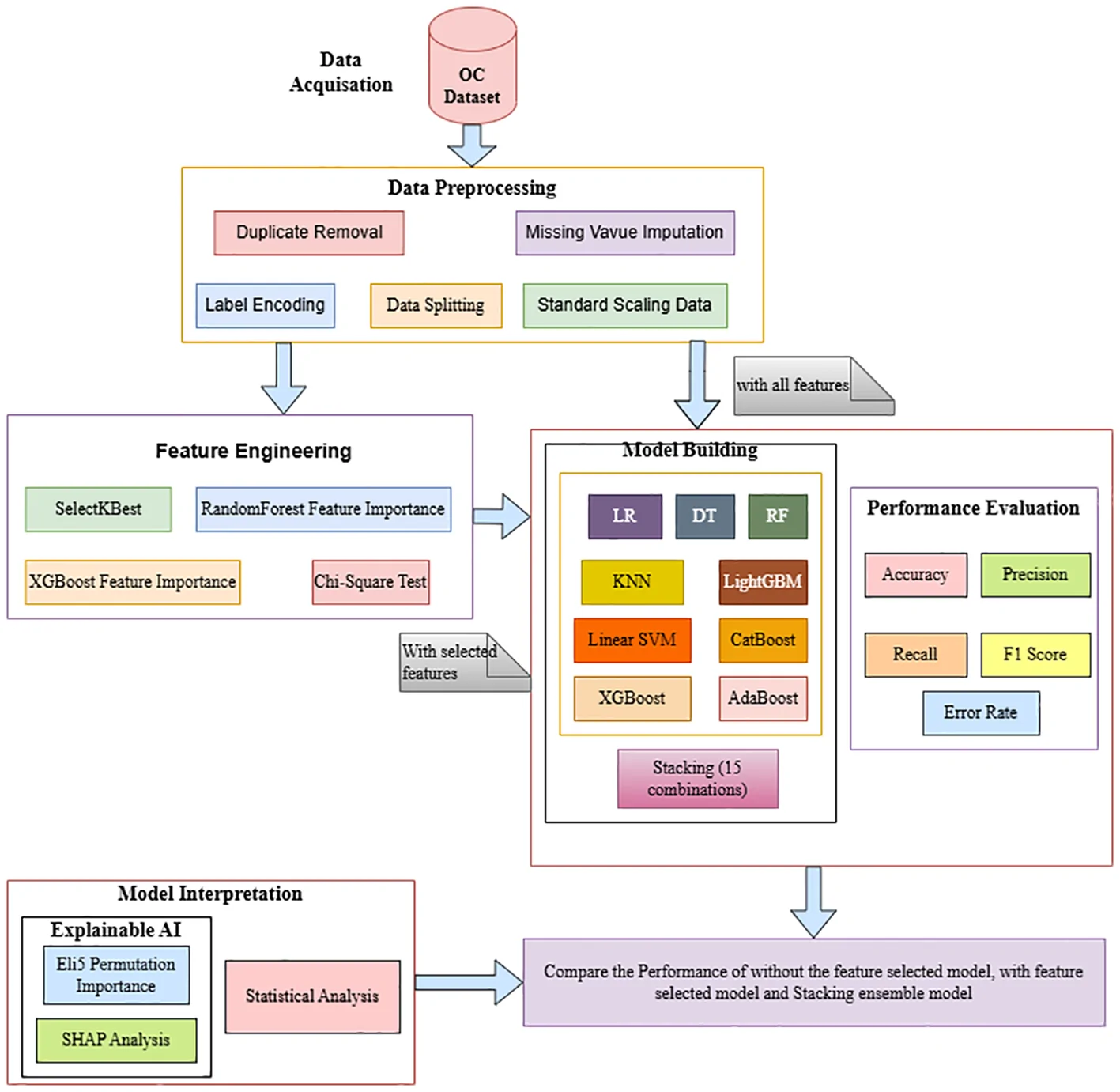
Multi stacking framework.

The second module, crucial for guaranteeing data quality for analysis and model readiness for use, is data preprocessing. This stage involves the treatment of missing data, outlier detection, encoding of categorical variables, and normalization of numerical ones. Secondly, the data set is divided into a training set and a test set for a fair assessment. These preprocessing steps help to ensure uniformity in the data, reduce noise, and optimize the data for effective machine learning analysis.

The third module combines a multi-method feature selection pipeline to decide which of the hundreds of variables are best to describe a healthy response. Four independent methods are used to create ranked lists of features: ANOVA (fclassif), Chi-square test, Random Forest feature importance, and XGBoost feature importance are all based on statistical significance, predictive relevance, and model-driven importance. The aggregation of these rankings will then be used to find out the top 20 consistent, consensus features. This mixed method ensures statistical stability of the model and its explicability with minimal risk of overfitting when dealing with high-dimensional medical data.

In the selected features of the fourth module, a range of machine learning models are created, such as classical models (Logistic Regression, Linear SVM, KNN, Decision Tree), ensemble models (Random Forest, AdaBoost) and boosting models (XGBoost, LightGBM, CatBoost). The performance of the model is comprehensively evaluated using accuracy, precision, recall, F1-score and area under the ROC curve (AUC) to get an overall measure of the model predictive ability. These findings show that either boosting algorithms have better discriminative performance, and additional performance improvement is attained by using a meta-learning approach.

The fifth module builds a model of stacking ensemble, in which the base learners are applied to make first-level predictions, which are then used by a meta-classifier to produce the final output. A number of stacking combinations are experimented with, and the pairwise combination of AdaBoost and the use of K-Nearest Neighbors comes out to be the most effective with the highest accuracy (0.7644) and better generalization performance when compared to individual models. This stacking structure takes advantage of the complementary strength of the constituent models, resulting in better predictive performance.

The latter element includes Explainable Artificial Intelligence (XAI) methods to guarantee the presence of clinical transparency and trust. The values of SHAP (SHapley Additive exPlanations), permutation importance, and cross-validated feature importance are used to measure the contribution made by each feature of the model output. The interpretability analyses focused exclusively on predictors retained after the leakage-control procedure, including pre-diagnostic questionnaire, demographic, screening, and biomarker variables. These insights enable more interpretation of the results, provide practical knowledge for clinicians about risk factors, and can be further applied in decision-making processes.

All five modules are integrated into a comprehensive and structured pathway leading to a clinically relevant, interpretable, and effective ovarian cancer prediction model. It is designed to be used in early detection and risk stratification programs, and is equipped with methodological rigor, state-of-the-art machine learning algorithms, and explainability.

### 3.1 Data source

The data for this study came from the Prostate, Lung, Colorectal, and Ovarian Cancer Screening Trial (PLCO), which is a large randomized screening trial performed in the United States by the National Cancer Institute. During the trial, from 1993 to 2001, about 155,000 people took part, and almost 78,000 women ages 55 to 74 were involved in the ovarian cancer arm of the trial. The women in the screening arm had yearly serum CA-125 testing and transvaginal ultrasound (TVU) testing, whereas the control arm received usual care. The study design was such that long-term follow-up of cancer incidence, tumor characteristics, treatment and cancer survival could be carried out.

The PLCO data is available via the Cancer Data Access System (CDAS). The data includes baseline clinical and demographic data (age at enrolment, BMI, reproductive history, family history), serial CA-125 levels and ultrasound values, and validated outcome measures (diagnosis, stage and mortality of patients with ovarian cancer). Diagnosis of ovarian cancer (yes/no) was the main outcome variable in this study. The dataset is class-imbalanced, as the number of patients with ovarian cancer is relatively small, needing special attention during processing and evaluation. PLCO’s prospective design, standardized screening protocol, and extensive clinical annotations make it a valuable tool for developing reliable and interpretable machine learning models for early prediction of ovarian cancer.

The PLCO dataset provides a rich and well-annotated resource for predictive modeling frameworks. It allows for a more comprehensive and multidimensional approach in the detection of ovarian cancer than the traditional biomarker-only or small cohort studies due to its wide spectrum of demographic, clinical and biomarker data.

#### 3.1.1 Study population and cohort selection

The initial arm of PLCO included 78,209 women, 77,691 without ovarian cancer and 518 with ovarian cancer. This made the number of patients with ovarian cancer very small (0.66% of the entire cohort), causing a substantial class imbalance. The study population and class distribution are summarized in **Table 1**.

**Table 1:** Study population and class distribution.

Dataset	Controls	Ovarian cancer	Total
Original PLCO	77,691	518	78,209
After random undersampling	518	518	1,036
Training (80%)	414	414	828
Testing (20%)	104	104	208

Machine learning algorithms trained on highly imbalanced data tend to overlook the minority class samples for good performance in detecting minority class samples. A solution to this problem is to undersample the majority class in a random way using the resample function from the scikit-learn library (random_state = 42, replace = False). All 518 ovarian cancer cases were kept and 518 control subjects were selected at random from the majority class.

This procedure yielded an analytically balanced sample of 1036 participants (518 with ovarian cancer and 518 controls). The balanced dataset was randomly shuffled, and then split into training (80%) and independent testing (20%) subsets with stratified sampling (random_state=42) to ensure balance in both subsets.

### 3.2 Data preprocessing

Strict preprocessing pipeline was adopted to warrant that the data that was used to model was precise and clinically valuable and analytically appropriate. The procedures were using the standard recommendations to the biomedical machine learning processes (31), and more specifically, the integrity of the variables reported in the PLCO data dictionary was preserved.

#### 3.2.1 Feature extraction and variable selection

Extracting features was initiated by the systematic review of all variables present in the ovar_prsn data and descriptions of these variables in the PLCO ovarian dictionary. The choice of variables relied on three principles:

1. Clinical relevance as used in the description of the PLCO documentation and earlier epidemiological studies on ovarian cancer;
2. Complete data, with variables that have the least missingness;
3. Developed correlations between reproductive history, screening outcomes, demographic factors and the risk of developing ovarian cancer.

After such evaluation, variables were obtained in several sections of the PLCO dictionary such as:

- Demographic factors (age, race, level of education) taken in Section 9-10;
- The results of screening tests in the form of CA-125 assay and transvaginal ultrasound (TVU) in Section 10: Screening;
- Section 22, Reproductive and family history variables, which include parity, age of menarche, hormone replacement therapy, and the first-degree family history of cancer;
- The diagnosing confirmation areas, location of cancer, and staging along with mortality data of Section 12: Cancer Diagnosis.

All the selected variables were categorized by cross-validation with the dictionary definition which was done to provide the appropriate understanding of the variables, accuracy of coding, and consistency among participants. With such a framework, the analytical data set that was produced was robust, retained patterns of clinical significance, and removed non-informative and redundant data.

#### 3.2.1 Class imbalance handling

The original PLCO cohort had a high degree of class imbalance with 77,691 control participants and 518 ovarian cancer cases. This imbalance can lead the machine learning algorithms to focus on the majority class and reduce the overall sensitivity of the algorithm to detect ovarian cancer. To address this problem, undersampling occurred randomly following leakage-aware preprocessing. In particular, 518 of the control subjects were randomly selected without replacement to be matched with the 518 ovarian cancer cases, providing a matched analytical cohort of 1,036. The balanced dataset was then split into independent training (80%) and testing (20%) sets, with a stratified sampling method (random_state = 42), making sure the class distributions were equalized. To eliminate majority class bias, while retaining the original distribution of the minority (ovarian cancer) class without adding synthetic observations, random undersampling was chosen.

#### 3.2.2 Missing data handling

Non-responding information was dealt with through a methodical and dictionary aware approach:

- Placeholders (as indicated by the PLCO codes) NaN (Not Applicable), NaN (No Form), NaN (Not Done (8)) and NaN (Unknown (9)) were changed to NaN to enable the statistical algorithms to treat them appropriately.
- Numerical data that was potentially skewed (such as CA-125 levels, biomarker concentrations) were replaced by the median, as suggested to fill in blood markers that were potentially skewed.
- Categorical variables (e.g., marital status, smoking history, screening categories) were imputed using the mode with the most common category that was clinically meaningful.

The measures decreased bias but did not alter the distribution of the data set.

#### 3.2.3 Categorical encoding

To make machine learning algorithms suitable to categorical variables, the variables were also coded with the corresponding coding methods, depending on the internal structure of the variables:

- Label Encoding was applied to the binary or ordinal data (e.g., yes/no data, screening trial flags).
- One Hot Encoding was used for the non-ordinal nominal variables such as race, education, and multiple-level screening results.

By this dual-encoding, the semantic meaning of categorical fields was retained, and no artificial ordering was added where it was not clinically present.

#### 3.2.4 Outlier identification

The clinical validation rules that were used to guide outlier detection were as stated in the PLCO documentation. Variables like the BMI, height, weight, and reproductive indicators have a certain code of Out of Range, which is mentioned on page 44 of the PLCO dictionary. Records with physiologically implausible flags or records that were against human values were not to be included in the analysis.

This step maintained the clinical accuracy and integrity of the data set by depending on the clinically proven thresholds and not arbitrary statistical cut-offs.

#### 3.2.5 Removal of data leakage variables

During preliminary feature selection and explainability analysis, certain variables demonstrated unusually high predictive power. Upon closer inspection, these variables were identified as post-diagnostic or follow-up variables that would not be available at the time of a real-world pre-diagnostic prediction. Their inclusion in model training constitutes data leakage, a methodological violation that artificially inflates model performance and undermines clinical validity.

Data leakage occurs when information that would not be available at the time of prediction is inadvertently incorporated during model training. In clinical prediction tasks, variables that encode mortality status, survival duration, or post-diagnosis follow-up inherently violate temporal causality. A model trained on such variables learns from outcomes rather than pre-diagnostic signals, producing optimistic performance estimates that cannot be reproduced in real-world deployment.

The following eight variables were identified and removed prior to any model training or feature selection:

- mortality_exitdays — number of days to mortality or study exit (post-outcome temporal marker)
- mortality_exitstat — mortality status at study exit (outcome-derived variable)
- mortality_exitage — age at mortality or censoring (post-event information)
- is_dead — binary mortality indicator (direct outcome proxy; classified as direct leakage)
- is_dead_with_cod — death with cause-of-death information (direct leakage)
- fstcan_exitage — age at first cancer diagnosis (post-event information)
- ovar_exitage — age at ovarian cancer exit (post-diagnosis indicator)
- ovariesr_f — ovarian cancer survival-related variable (post-diagnostic)

Among these, is_dead and is_dead_with_cod were classified as direct leakage variables because they explicitly encode the study outcome. The remaining six variables constitute indirect leakage, as they capture temporal endpoints or follow-up durations that are only observable after cancer diagnosis or mortality. Although these variables exhibit strong statistical associations with the outcome, none of them would be available in a real-world screening scenario at the point of risk assessment. Their inclusion would lead to false inflated predictive performance and to the loss of the clinical credibility of the framework.

All eight variables were excluded prior to feature selection, model training and explainability analysis. After this removal, baseline demographic, screening, questionnaire-derived and biomarker measures were used in all models, without the inclusion of the cancer diagnosis. This allows reporting of true pre-diagnostic prediction data, and not *post-hoc* inferences from data related to outcomes.

This leakage aware pre-processing step is an important methodological contribution of this work. This approach acknowledges, records and eliminates variables that can leak through the cracks before any analytical step, which is often omitted in published ovarian cancer ML studies. This yields a more conservative, but methodologically sound and clinically repeatable prediction model, which could be translated to real-life ovarian cancer screening and risk stratification programs.

#### 3.2.6 Definition of prediction time point

The goal of the proposed framework is to make an estimate of ovarian cancer risk, based on information prior to clinical diagnosis. Variables that directly or indirectly carry post-diagnostic information are excluded and the prediction index is defined as the most recent screening record prior to the onset of ovarian cancer, or prior to censoring.

Before the diagnosis of ovarian cancer, only variables available for measurement prior to the diagnosis were included in the models. Other variables describing mortality outcomes, survival time, age at cancer diagnosis, post-diagnostic follow-up, and other information that was temporally downstream were excluded in the pre-processing phase to minimize information leakage. As a result, the proposed framework calculates ovarian cancer risk based on pre-diagnostic demographic characteristics as well as on questionnaire items and screening variables and cancer biomarker measurements obtained prior to outcome ascertainment.

The proposed model is not a predictive model for a specific time but a pre-diagnostic risk prediction model. The prediction horizon was thus set as the time before diagnosis of ovarian cancer when the predictors selected were available in the PLCO screening program. The aim was to detect people who were at higher risk based on information that is available before becoming diagnosed with cancer, not to foretell the likelihood of someone developing cancer within a set timeframe (such as a certain number of months or years).

#### 3.2.7 Data splitting

The processed data was divided into 80/20 training and testing sets. A stratified sampling approach was used so as to retain the initial proportion of ovarian cancer cases (positive class) and non-cases (negative class). This was necessary as there existed an imbalance between the classes as was the case with ovarian cancer datasets, and also because the model has to be able to generalize properly between the two groups.

#### 3.2.8 Leakage-aware analytical pipeline

A leakage-aware analytical workflow was used for the development of the model, to prevent information leakage. Explicit outcome-derived variables, such as diagnosis status, treatment variables, indicators of mortality, survival duration and other post-diagnostic variables, were excluded from the original data set. Random undersampling of the majority class was then performed on the rest of the data set, and it was randomized. After that, the balanced dataset was split into independent training (80%) and testing (20%) sets with stratified sampling (random_state = 42) to ensure that the class distributions were kept equal.

Only the training data set was used for model development. The feature selection, model training, hyperparameter optimization, and ensemble construction were not done using the independent test set. The test set was only used for final performance evaluation to ensure that the proposed framework is evaluated objectively.

**Figure 2**. Proposed ovarian cancer prediction framework analytical pipeline with leakage aware. Only the training data was used to fit all data-driven preprocessing steps such as missing-value imputation, categorical encoding, scaling/normalization, and feature selection. The transformations obtained were then applied to the independent test set, without refitting or test set information. The pipeline starts with the original PLCO ovarian screening cohort, then leakage variables are explicitly removed, class balancing via random undersampling, train–test splitting based on strata, multi-method feature selection, machine learning model development, independent model evaluation and explainability analysis. The test set used was an independent test set, which was used only for the final evaluation, thus reducing the optimistic bias.

**Figure 2:**
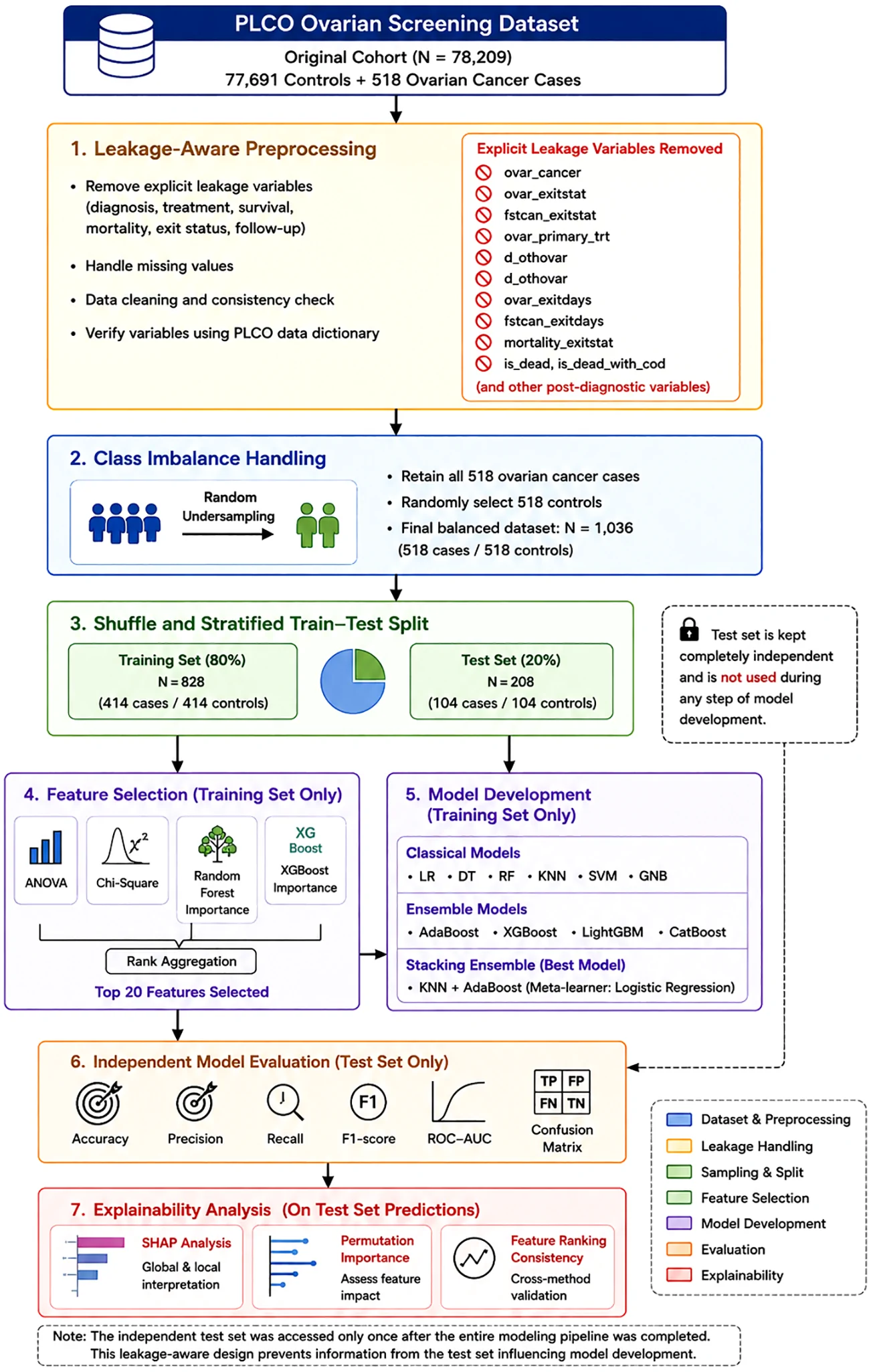
Leakage-aware pipeline.

### 3.3 Feature selection

There are many heterogeneous variables in clinical datasets, including the demographic, behaviors, screening related, and diagnostic; as in the case of PLCO, many of these variables are interrelated or lowly tied to the clinical outcome. A multi stage feature selection framework was employed to stabilize the model, reduce overfitting, and enhance interpretability. It is a methodology that integrates filter-based, and embedded, to determine the most information-rich predictors to classify ovarian cancer.

#### 3.3.1 Filter-based selection

Filter method was applied as a preliminary screening mechanism to determine the variables which had statistically significant relationship to the target outcome. Two supplementary methods were used:

• ANOVA F-test (f_classif):

This test measures the level of both the numerical features that distinguish the two outcome groups (ovarian cancer vs. no cancer). The features with high F-scores mean a high discriminatory power.

The F-score (ANOVA) of each feature is calculated as shown in Equation (1)

(1) $$F=\frac{Between-group\;variance}{Within-group\;variance}$$

• Chi-square test:

The Chi-square test of statistical dependence is applied to categorical variables to determine the statistical dependence of each predictor and the outcome. The high Chi-square variables are more likely to be relevant for classification. The Chi-square statistic is calculated as shown in Equation (2).

(2) $$\chi^{2}=\sum \frac{{\left(O-E\right)}^{2}}{E}$$

Where:

• O = observed frequency

• E = expected frequency

These filter approaches gave a preliminary sign of feature relevance without any machine learning model. They also assisted in the removal of noisy or weakly correlated variables, before proceeding to more complex kinds of selections.

#### 3.3.2 Embedded selection

To encode the insights of the model, embedded feature selection was implemented with the help of tree-based machine learning algorithms, an inherent measure of feature significance, which in turn quantify feature importance in training:

• Random Forest importance:

Random Forests are used to quantify the decrease in impurity added by each feature by many decision trees. This methodology does capture nonlinear connections and interactions that might not be detected in statistical analysis. The Random Forest feature importance is calculated as shown in Equation (3).

(3) $$Importance\left(X_{j}\right)=\sum \Delta Gini$$

Where ΔGini = decrease in Gini impurity with the use of the feature to split.

• Gradient Boosting and XGBoost importance:

The algorithms used in boosting create decision trees dedicated to hard samples. The combined profit and bifurcated strength give strong measures of feature effect. The XGBoost feature importance based on gain is calculated as shown in Equation (4).

(4) $$Gain\;X_{j}=Improvement\;in\;model\;Accuracy\;\text{When}\;X_{j}\;is\;used\;for\;splitting$$

These intrinsic techniques are indicative of the behavior of features in real predictive models and in many cases they detect interaction-driven predictors which are not apparent using purely statistical techniques.

Rank Aggregation: All four of the selection strategies (ANOVA, Chi-square, Random Forest importance and XGBoost importance) were followed by a rank aggregation. Attributes that kept on reoccurring in the highest ranking in the different approaches were given first preference. This resulted in the selection of a beautiful set of 20 most informative predictors that was used for training and testing the machine learning models. For each feature *j*, the aggregate rank was calculated by summing its individual ranks obtained from the four feature-selection methods, as shown in Equation (5).

(5) $$R_{j}=R_{j,\;ANOVA}+R_{j,\chi^{2}}+R_{j,RF}+R_{j,\;XGB}$$

where $R_{j,\;ANOVA}$, $R_{j,\chi^{2}}$, $R_{j,RF}$, and $R_{j,\;XGB}$ represent the rank assigned to feature *j* by ANOVA F-test, Chi-square test, Random Forest feature importance, and XGBoost feature importance, respectively. Features with the lowest aggregate rank *R_j_* were considered the most consistently important and were prioritized for selection.

This multi-method approach guaranteed:

- robust feature stability,
- better model interpretability,
- reduced multicollinearity, and
- improved predictive behavior.

In order to make the selected predictors more clinically relevant, the main variables selected by the proposed multi-method feature selection framework are summarized in **Table 2**. Official PLCO variable definitions, the clinical categories, the context in which the variables are measured, and leakage assessment are listed in the table. All chosen variables were checked against the official PLCO data dictionary to ensure they represented data prior to the ascertainment of the outcome and not information related to outcome or survival. This verification helps the design of the proposed prediction framework be leakage-aware.

**Table 2:** Clinical description of key PLCO variables used in the proposed framework.

Variable	Official PLCO description	Category	Available before diagnosis	Leakage assessment
ovary_trial_flag	Indicates whether the participant had ovaries at baseline (0 = No, 1 = Yes).	Baseline eligibility	Yes	No
entryage_sqx	Age of the participant at SQX analysis entry.	Demographic	Yes	No
entrydays_sqx	Number of days from randomization until completion of the Baseline Questionnaire (BQ) and Supplemental Questionnaire (SQX).	Screening timeline	Yes	Low
ph_ovar_sqx	Personal history of ovarian, fallopian tube, or primary peritoneal cancer prior to SQX analysis entry (0 = No, 1 = Yes).	Medical history	Yes	No*
ph_ovar_dhq	Personal history of ovarian, fallopian tube, or primary peritoneal cancer prior to DHQ analysis entry (0 = No, 1 = Yes).	Medical history	Yes	No*
ph_any_dhq	Personal history of any cancer before DHQ analysis entry.	Medical history	Yes	No
ca125_result5	Result of the fifth CA-125 screening measurement.	Biomarker	Yes (if measured before diagnosis)	NO

* Note that the variables ph_ovar_sqx and ph_ovar_dhq are participant-reported personal history items that precede the analysis entry that corresponds to the respective SQX/DHQ. As they were obtained as pre-diagnostic variables, they were not used as outcome variables but retained. All the retained serial CA-125 values were verified to have been measured prior to the established prediction/index time for the relevant participants and were therefore considered to be screening predictors prior to the diagnosis.

### 3.4 Model development

The implementations of a large set of machine learning algorithms were used to create a strong predictive model of ovarian cancer based on the PLCO dataset (32). The choice of the models involved the classical machine learning algorithms and sophisticated ensemble based methods, guaranteeing a high level of exploration of the linear, nonlinear, and tree based decision boundaries.

The following classifiers were considered:

1. Support Vector Classifier (SVC)

Operational in high dimensional space and in a position to deal with non-linear boundaries by doing kernel transformations. (33, 34)

2. K-Nearest Neighbors (KNN)

A distance-based classifier that is useful for exploratory benchmarking and that captures local neighborhood structure.

3. Decision Tree Classifier

Provides understandable decision ways that are rule based and have hierarchy.

4. Random Forest Classifier

A set of decision trees trained on bootstrapped samples and averaged.

5. Bagging Classifier

Bootstrap aggregation offers stability of variance and robustness of the classifiers for heterogeneous data. (35)

6. Gradient Boosting Classifier

Trains sequential weak learners to minimize the residual errors for good performance on structured clinical data.

7. XGBoost Classifier

The most efficient use of gradient boosting that is regularized, and can identify intricate interplay between predictors.

8. CatBoost Classifier

A model that is not susceptible to overfitting has been designed for categorical data and can perform better in feature space, where the data is a mixture of data types.

9. Voting Classifier (Soft and Hard Voting)

Combines predictions of multiple base learners and offers a basic but effective ensemble approach.

#### 3.4.1 Stacking ensemble learning

Since clinical datasets usually have a combination of numerical, categorical, screening, and diagnostic variables, one classifier might not be able to represent all patterns. To solve this heterogeneity, the use of an ensemble learning framework was adopted to take advantage of the complementary abilities of varying algorithms.

The tree-based models (Random Forest, XGBoost, and CatBoost) are better at learning (36) non-linear interaction and automatically processing feature importance; meanwhile, models based on the margins (like SVC) are better at maximizing the separation between classes (37). The distance based models (KNN) bring a local structural information. Ensuring that these different views are combined together in ensemble learning increases the quality in predicting, stability, and interpretability.

A soft voting assembly was built on the base learners with the highest performance when measured separately on each model. The weighted average of the class probabilities is used to obtain the final prediction in soft voting, and this allows the ensemble to use strong probabilistic evidence between models (38). The method causes less model variance, less overfitting, and greater robustness between patient subgroups.

A stacking ensemble learning framework was designed to enhance the robustness of the prediction model, by combining various combinations of heterogeneous machine learning algorithms. The base learners evaluated were: Logistic Regression (LR), Random Forest (RF), K-Nearest Neighbors (KNN), Linear Support Vector Machine (SVM), and AdaBoost. The optimum architecture of the ensemble was explored by testing a number of stacking configurations of two, three and five models.

The classifiers were created for each of the stacking models using the Stacking Classifier class from the scikit-learn package. For all stacking combinations, the common meta-learner used was Logistic Regression (random_state = 42). In training the models, we implemented five-fold cross-validation (cv = 5) to obtain out-of-fold predictions from the base learners to train the meta-learner. This process was used to ensure that information would not leak from the base-learning to the meta-learning phase.

The parameter passthrough=False was used because the meta-learner was given the predictions from the base classifiers instead of the base classifiers’ feature set. Only the training set was used to train all ensemble models and the independent test set was used only for the final evaluation. The metrics used for comparing the performance of the evaluated stacking configurations were Accuracy, Precision, Recall, F1-score, Error Rate, ROC–AUC and confusion matrices.

**Figure 3**. Architecture of the planned stacking ensemble learning structure. The out-of-fold predictions were obtained by running multiple heterogeneous machine learning models on 5-fold cross validation, where a Logistic Regression meta-learner was used to combine the out-of-fold predictions and get a final prediction. Independent test data set is separated till the final test stage.

**Figure 3:**
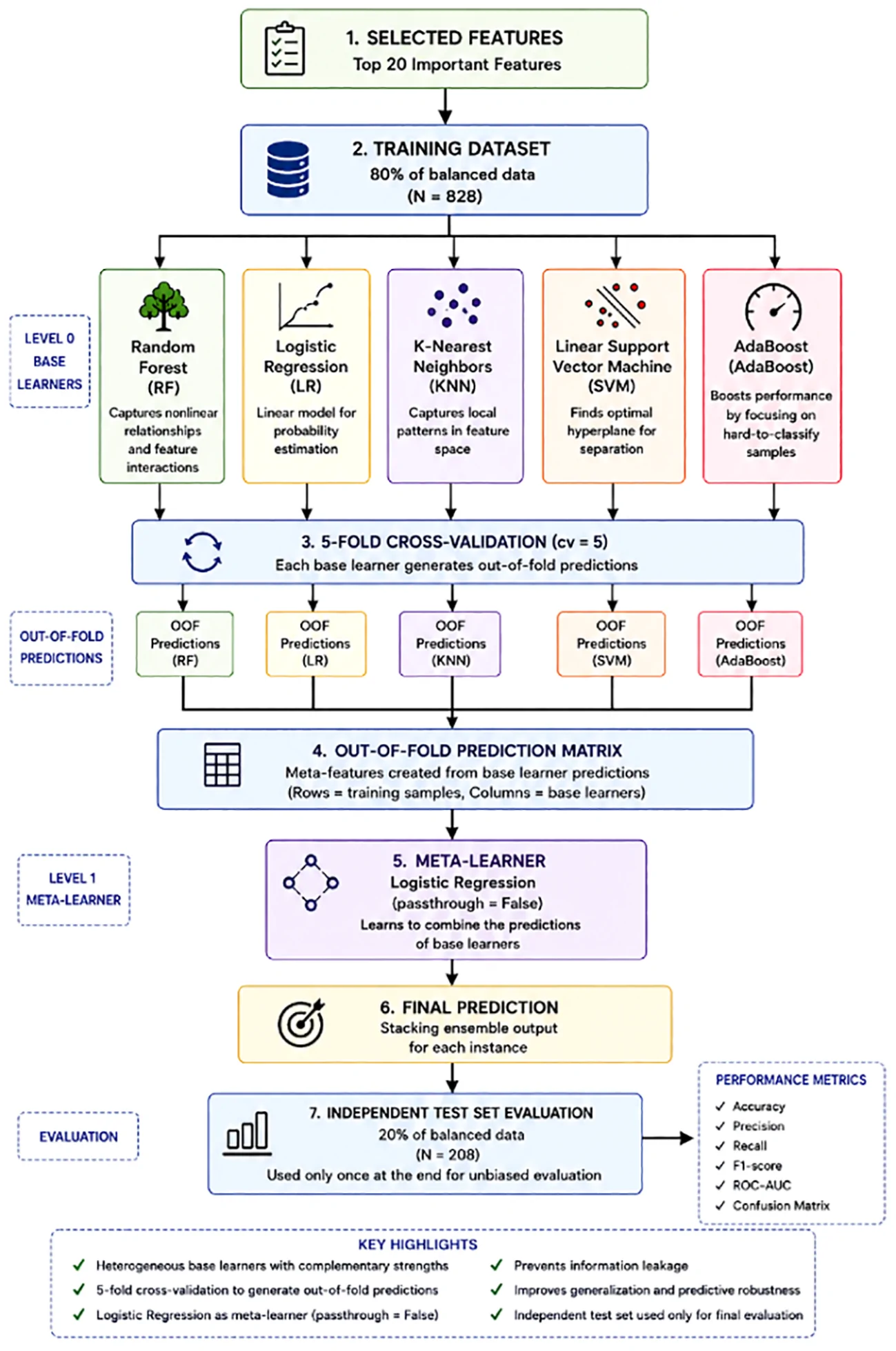
Proposed stacking ensemble learning framework.

### 3.5 Performance evaluation

Both the traditional machine learning performance metrics and clinically relevant diagnostic metrics were used to evaluate model performance. The accuracy, precision, recall, F1-score, error rate, receiver operating characteristic area under the curve (ROC–AUC) and confusion matrix were used as conventional metrics. Other diagnostic metrics such as Sensitivity, Specificity, Positive Predictive Value (PPV), Negative Predictive Value (NPV), Balanced Accuracy and Matthews Correlation Coefficient (MCC) were also calculated for a more clinically meaningful evaluation of ovarian cancer prediction, as were precision–recall area under the curve (PR–AUC).

The Precision, Recall and F1-Score values given in the comparative model-performance tables are weighted-average values, computed over both classes unless stated otherwise. Furthermore, the Precision, Recall and F1-score values for the best-performing model are also provided for each class (control and ovarian cancer) for clinical interpretation.

#### 3.5.1 Calibration and clinical utility analysis

The best-performing KNN–AdaBoost stacking model was further tested with regard to calibration and clinical utility. The calibration curve was used to assess the calibration of the model along with the Brier score, the calibration intercept, and the calibration slope. Decision Curve Analysis (DCA) was conducted to determine net benefit of the model at various rates of risk, and to compare it to the treat-all and treat-none strategies. The DCA was performed on the predicted probabilities, which were adjusted for the original prevalence of ovarian cancer in PLCO by random undersampling of the analytical cohort.

### 3.6 Model configuration and reproducibility

All experiments were conducted using Python 3.13.5 in an Anaconda-based 64-bit environment. The analyses used NumPy 2.1.3, Pandas 2.2.3, scikit-learn 1.6.1, SHAP 0.52.0, XGBoost 3.0.4, LightGBM 4.6.0, and CatBoost 1.2.8. A fixed random seed of 42 was used wherever applicable to ensure reproducibility. The model configurations and hyperparameters are provided in **Supplementary Table 1**.

## 4 Results

### 4.1 Baseline model performance

All machine learning models were initially trained on the full feature set, before any feature selection or dimensionality reduction methodology is applied to create a baseline on ovarian cancer classification. This baseline analysis offers the information about the ability of every algorithm to work with the raw and high-dimensional PLCO data and can be used to highlight the models which have a high intrinsic discriminatory power.

The baseline accuracy, precision, recall, F1-score and error rate of each classifier is presented in **Table 3**.

**Table 3:** Model performance comparison table.

Model	Accuracy	Precision	Recall	F1 Score	Error rate
Logistic Regression	0.663462	0.663522	0.663462	0.663430	0.336538
Linear SVM	0.687500	0.687656	0.687500	0.687435	0.312500
K-Nearest Neighbors	0.538462	0.538476	0.538462	0.538419	0.461538
Random Forest	0.716346	0.716526	0.716346	0.716287	0.283654
LightGBM	0.735577	0.735773	0.735577	0.735522	0.264423
Decision Tree	0.677885	0.680708	0.677885	0.676621	0.322115
AdaBoost	0.764423	0.767415	0.764423	0.763762	0.235577
CatBoost	0.750000	0.750370	0.750000	0.749908	0.250000

The performance of looking at the number of key trends between classifiers in the leakage-free model at baseline, as seen in **Figure 4**, shows the differences between the two models. That’s because of the existence of boosting-based ensemble models and the best among them is AdaBoost with accuracy of 0.7644 followed by CatBoost (0.7500) and LightGBM (0.7356), which is a good fit for this data. The results show interferential error correction and weighted learning schemes inherent in boosting algorithms are appropriate to describe nonlinear relationships in structured screening and the questionnaire data. Random Forest has shown competitive performance (0.7163 accuracy), and it is true that tree-based ensembles are powerful in the modeling of complex feature interactions.

**Figure 4:**
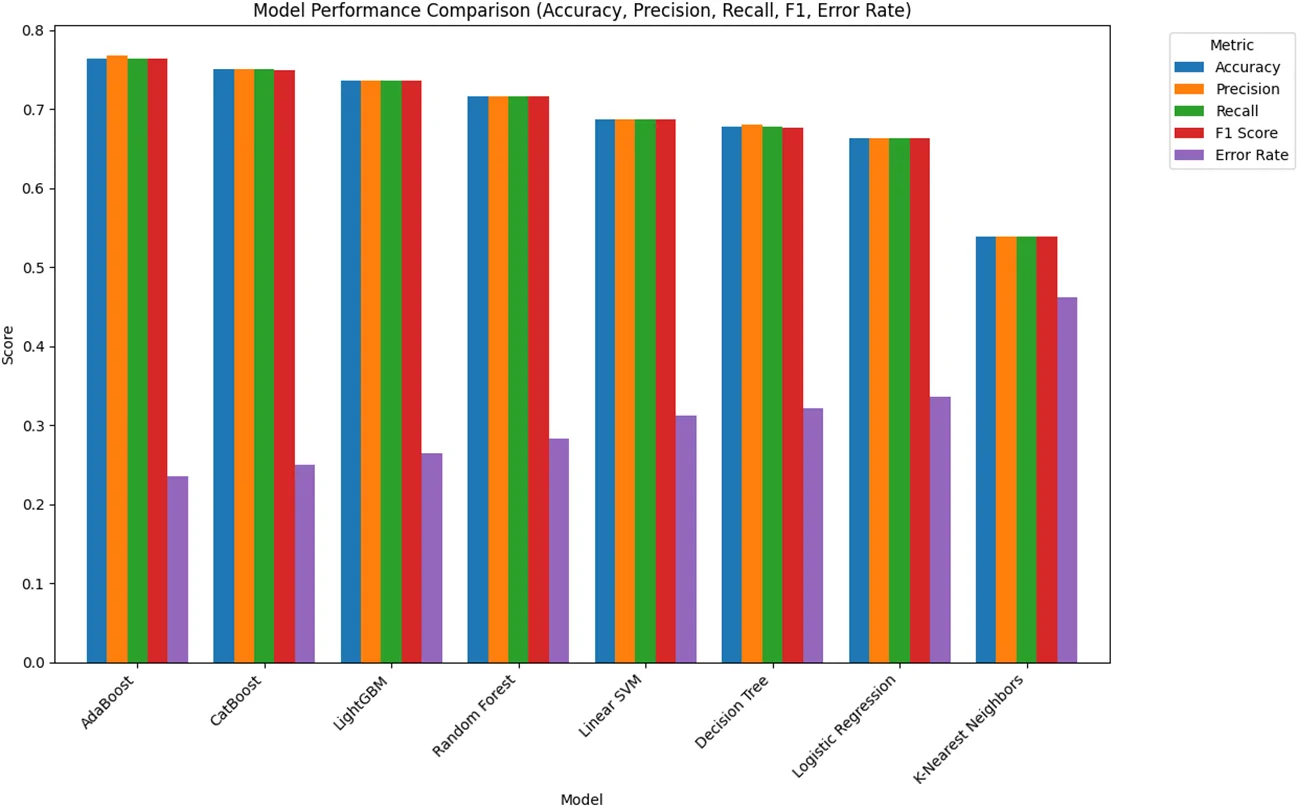
Model performance: comparison plot.

However, Linear models like Linear SVM (0.6875) and Logistic Regression (0.6635) performed moderately, which means that there is some linear separability in the feature space, but nonlinear model training has a better discriminative ability. Decision Tree classifier (0.6779) demonstrated fairly good results but performed worse in comparison with ensemble techniques presumably because it can be overfitted and suffers a depth-based overgeneralization.

Naturally, K-Nearest Neighbors (0.5385) had the lowest accuracy, which is associated with its sensitivity to feature scaling, mixed type of variables and high-dimensional structure. The distance-based approaches tend to be challenging in situations where informative features are spread unevenly across non-homogeneous clinical variables.

In general, the base line analysis indicates that the best predictive accuracy in this leakage free environment is obtained with boosting and ensemble based classifier. Nevertheless, the variability of observed performance among the models highlights the necessity of additional optimization via a feature selection phase, the stacking of ensembles, and hyperparameter optimization to enhance stability and generalization.

### 4.2 Feature selection results

#### 4.2.1 Ranking across multiple methods

Multi-method feature selection was employed in order to focus on the high-dimensional PLCO data and determine the most clinically and statistically significant predictors. **Table 4**, Ranking of Features, demonstrates that four complementary methods were applied to assess the strength of association between each feature and the ovarian cancer outcome independently in ANOVA (fclassif), Chi-square, Random Forest feature importance, and XGBoost feature importance.

**Table 4:** Ranking of features.

Features	f_classif_Rank	chi2_Rank	RandomForest_Rank	XGBoost_Rank	Sum_of_Ranks
ph_ovar_sqx	1.0	4.0	1.0	1.0	7.0
ph_ovar_dhq	2.0	7.0	14.0	5.0	28.0
ph_any_dhq	12.0	6.0	12.0	6.0	36.0
entrydays_sqx	21.0	2.0	2.0	11.0	36.0
ca125_result5	10.0	5.0	21.0	12.0	48.0
ovary_trial_flag	3.0	38.0	8.0	3.0	52.0
entryage_bq	8.0	21.0	17.0	8.0	54.0
entryage_sqx	18.0	41.0	4.0	2.0	65.0
entrydays_dhq	19.0	1.0	6.0	42.0	68.0
bq_age	9.0	22.0	13.0	38.0	82.0
ovar_mra_stat0	5.0	9.0	56.0	22.0	92.0
age	11.0	23.0	15.0	48.0	97.0
sisters	38.0	30.0	26.0	18.0	112.0
ph_any_sqx	31.0	17.0	3.0	77.0	128.0
ca125_result4	14.0	8.0	45.0	61.0	128.0
bcontrt	25.0	14.0	35.0	58.0	132.0
entryage_dhq	15.0	32.0	18.0	71.0	136.0
ca125_result3	37.0	15.0	61.0	25.0	138.0
ovar_eligible_dhq	4.0	16.0	36.0	87.0	143.0
tvu_result0	20.0	11.0	39.0	75.0	145.0

Both procedures produced a ranked list of variables in terms of model-driven importance or statistical significance. The aggregation of these rankings has been done using a sum-of-ranks strategy which enables the identification of the most strongly consistent features of the methods. This ensemble-ranking method minimized the bias of any individual selection method and gave the chance that both linear and non-linear relationships would be identified.

The combined ranking (sum of ranks) identified the following as the Top 20 most influential predictors:

Top Features (Across Methods):

Ph_ovar_sqx, ph_ovar_dhq, ph_any_dhq, entrydays_sqx, ca125_result5, ovary_trial_flag, entryage_bq, entryage_sqx, entrydays_dhq, bq_age, ovar_mra_stat0, age, sisters, ph_any_sqx, ca125_result4, bcontrt, entryage_dhq, ca125_result3, ovar_eligible_dhq, tvu_result0

These predictors represent a multidimensional combination of screening biomarkers (multiple CA-125 measurements and TVU findings), demographic and age-related indicators, questionnaire-derived gynecological and symptom-related variables, screening participation markers, reproductive history, and family risk factors. Notably, no outcomes were used as mortality or post-diagnostic variables, which makes the model present the realistic pre-diagnostic prediction situation.

Once the top 20 features were chosen, the final training and testing sets were of the following dimension:

- X_train_selected: (828, 20)
- X_test_selected: (208, 20)

This confirms that the feature selection mechanism effectively reduced the high dimensional data to the most useful elements. This dimensionality reduction enhanced the computational efficiency, reduced redundancy, diminished the risk of overfitting, and increased the clinical interpretability and generalizability of the predictive framework.

### 4.3 Model performance after feature selection

On several model performances, feature selection enhanced performance. The new metrics are indicated in **Table 5**.

**Table 5:** Model performance after feature selection.

Model	Accuracy	Precision	Recall	F1 Score	Error rate
AdaBoost (Selected)	0.745192	0.746308	0.745192	0.744903	0.254808
XGBoost (Selected)	0.740385	0.740385	0.740385	0.740385	0.259615
CatBoost (Selected)	0.735577	0.736123	0.735577	0.735424	0.264423
LightGBM (Selected)	0.730769	0.730769	0.730769	0.730769	0.269231
Random Forest (Selected)	0.725962	0.727667	0.725962	0.725448	0.274038
Linear SVM (Selected)	0.711538	0.716667	0.711538	0.709821	0.288462
Decision Tree (Selected)	0.711538	0.712245	0.711538	0.711298	0.288462
Logistic Regression (Selected)	0.692308	0.693452	0.692308	0.691852	0.307692
K-Nearest Neighbors (Selected)	0.605769	0.605769	0.605769	0.605769	0.394231

After the retraining using the chosen 20 leakage-free features, the performance patterns show that ensemble-based boosting algorithms are the most effective classifiers. AdaBoost performed the most accurately (0.7452) and then XGBoost (0.7404), CatBoost (0.7356), and LightGBM (0.7308), which proves that boosting techniques are especially adequately placed to identify nonlinear interactions among structured screening and questionnaire-based variables. Also, Random Forest (0.7260) did not show any significant changes, which indicates that the tree-based ensemble can be utilized to efficiently model complex relationships between features even when dimensionality reduction is performed (39). The reduced accuracy relative to the previous experiments is a sign of elimination of leakage-prone variables and is a more realistic evaluation of actual pre-diagnostic predictive accuracy.

The less effective linear models (Linear SVM 0.7115 and Logistic Regression 0.6923) still revealed some moderate effectiveness, implying that some linear separability can be observed in the predictors considered but nonlinear methods of risk enumeration are more effective due to the multidimensionality of ovarian cancer. Decision Tree model (0.7115) was also similar to the Linear SVM but weaker than the ensemble method probably because of its low generalization ability. Predictably, K-Nearest Neighbors (0.6058) had the lowest accuracy, which still indicated the existing sensitivity of distance-based models to feature distributions of different types. In summary, the feature set selected was clinically meaningful, preserved clinically relevant predictive information, improved model validity, and boosting algorithms were found to be the most robust classifiers in the leakage-free setting.

### 4.4 ROC–AUC analysis

ROC–AUC values have been calculated for both training and testing sets in a threshold independent manner.

#### 4.4.1 AUC (training data)

ROC curves for training data after feature selection are clearly represented in the diagram in **Figure 5**. **Table 6** gives the AUC scores for training data after feature selection.

**Figure 5:**
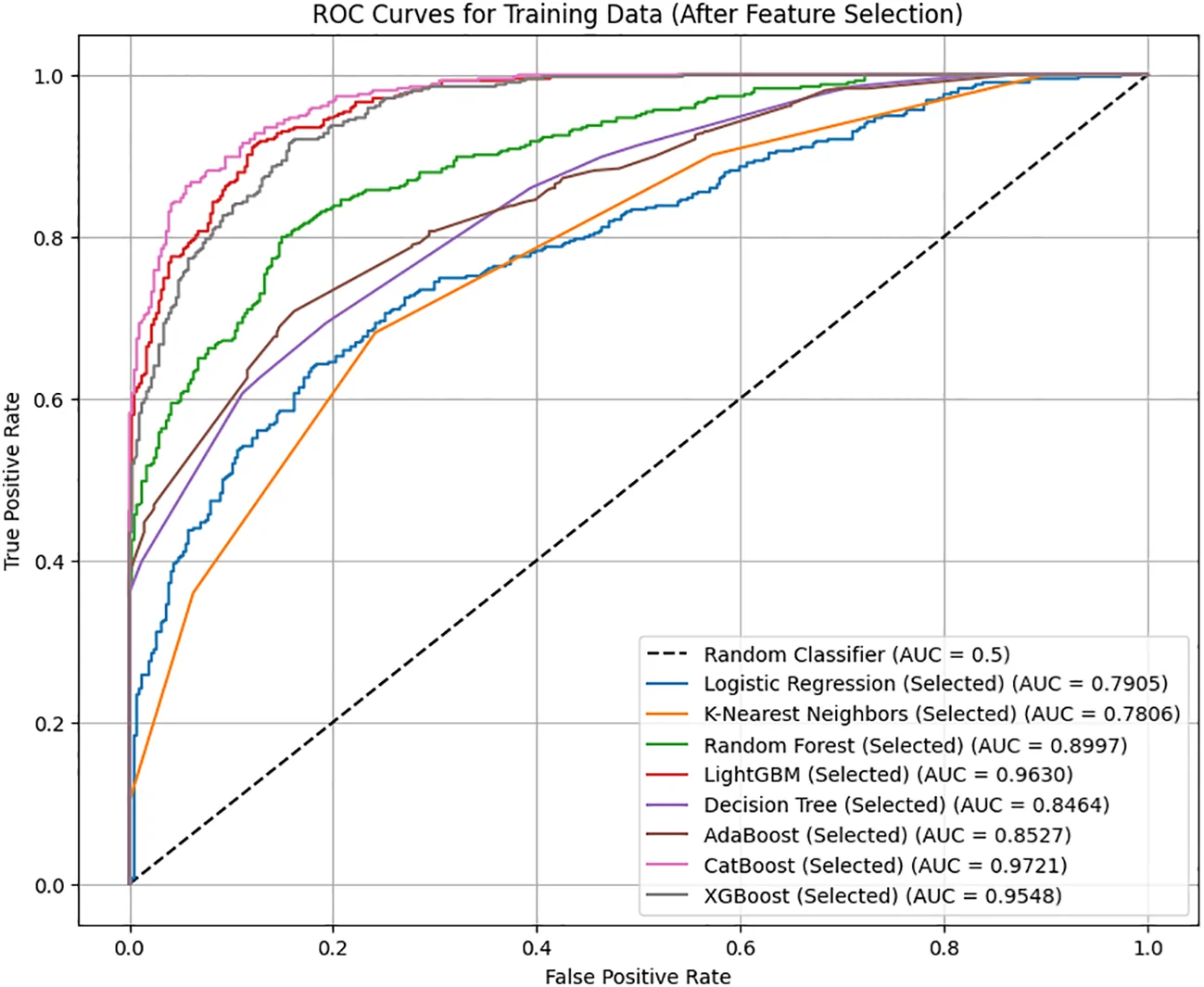
ROC curves for training data (after feature selection).

**Table 6:** AUC Scores for training data (after feature selection).

Model	AUC
CatBoost (Selected)	0.972082
LightGBM (Selected)	0.963042
XGBoost (Selected)	0.954844
Random Forest (Selected)	0.899653
AdaBoost (Selected)	0.852730
Decision Tree (Selected)	0.846423
Logistic Regression (Selected)	0.790526
K-Nearest Neighbors (Selected)	0.780575

#### 4.4.2 AUC (testing data)

The generalization performance was strong, especially for boosting models. **Figure 6** represents ROC curves of test data following feature selection. Test data AUC scores post feature selection are in **Table 7**.

**Figure 6:**
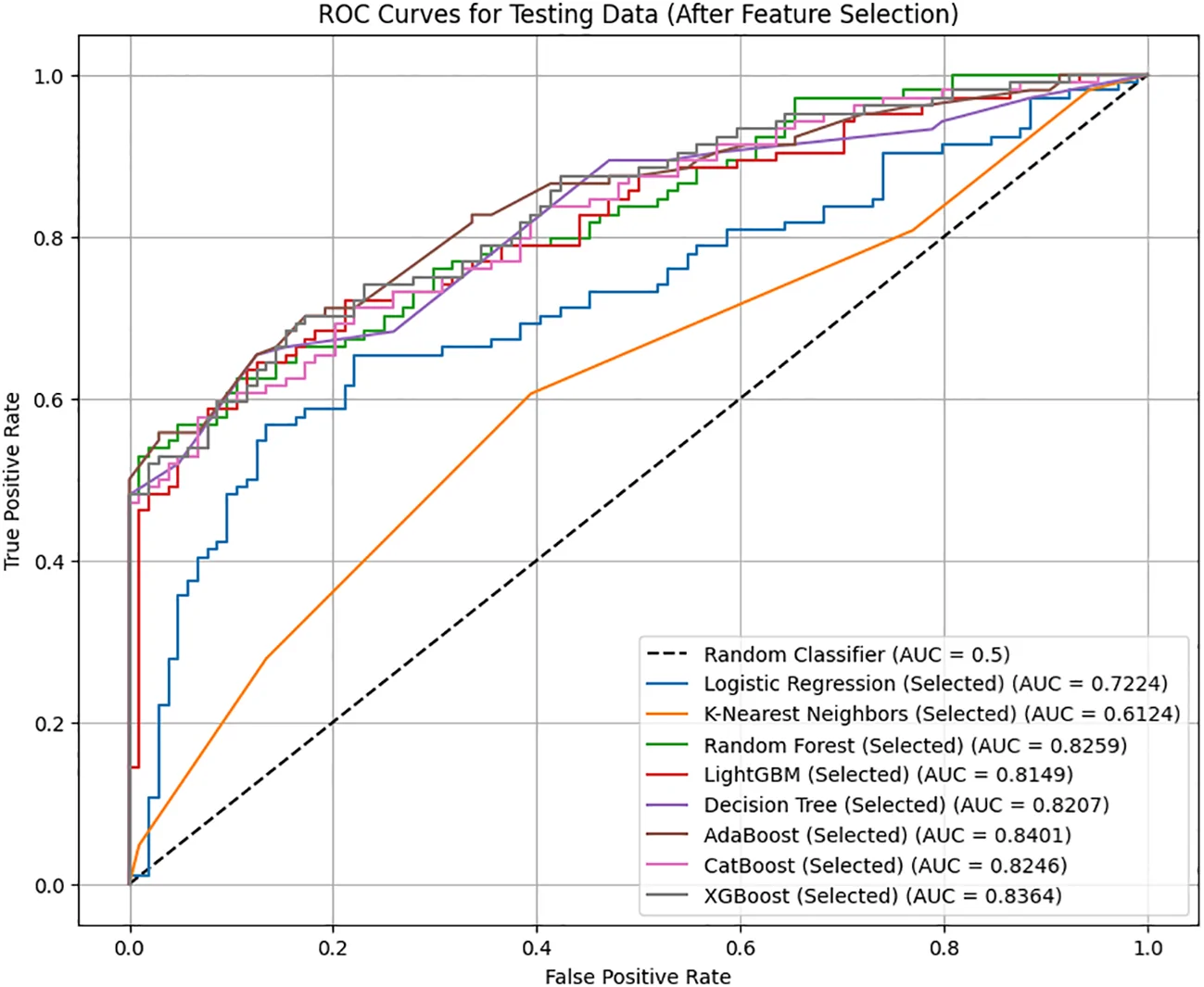
ROC curves for testing data (after feature selection).

**Table 7:** AUC scores for testing data (after feature selection).

Model	AUC
AdaBoost (Selected)	0.840144
XGBoost (Selected)	0.836354
Random Forest (Selected)	0.825906
CatBoost (Selected)	0.824612
Decision Tree (Selected)	0.820729
LightGBM (Selected)	0.814904
Logistic Regression (Selected)	0.722356
K-Nearest Neighbors (Selected)	0.612426

Boosting models provide an exceptionally good generalization properties in predicting ovarian cancer on the PLCO data.

### 4.5 Stacking ensemble results

In order to further improve the predictive performance compared to that of the individual classifiers, several stacking ensemble architectures were tested (40). Stacking builds on the merits of heterogeneous base learners, in the sense that it enables a meta-classifier to trade off optimal combinations of their forecasts. It is specifically useful in cases where a clinical dataset like PLCO is involved, and the variables can have complicated relationships which different algorithms can model in different ways (**Table 8**) gives the performance of stacking model following feature selection.

**Table 8:** Stacking model performance after feature selection.

Model	Accuracy	Precision	Recall	F1 Score	Error rate
Stacking (KNN + AdaBoost)	0.764423	0.770041	0.764423	0.763192	0.235577
Stacking (RF + SVM)	0.735577	0.738242	0.735577	0.734835	0.264423
Stacking (RF + KNN + SVM)	0.735577	0.738242	0.735577	0.734835	0.264423
Stacking (LR + RF + SVM)	0.730769	0.732923	0.730769	0.730145	0.269231
Stacking (RF, SVM, KNN, LR, AdaBoost)	0.730769	0.732923	0.730769	0.730145	0.269231
Stacking (RF + AdaBoost)	0.725962	0.726990	0.725962	0.725651	0.274038
Stacking (LR + AdaBoost)	0.721154	0.726515	0.721154	0.719494	0.278846
Stacking (RF + KNN)	0.716346	0.717979	0.716346	0.715814	0.283654
Stacking (SVM + AdaBoost)	0.716346	0.722286	0.716346	0.714439	0.283654
Stacking (LR + RF + KNN)	0.716346	0.717331	0.716346	0.716025	0.283654
Stacking (RF + LR)	0.711538	0.712245	0.711538	0.711298	0.288462
Stacking (LR + SVM)	0.711538	0.716667	0.711538	0.709821	0.288462
Stacking (KNN + SVM)	0.711538	0.715443	0.711538	0.710226	0.288462
Stacking (LR + KNN + SVM)	0.711538	0.715443	0.711538	0.710226	0.288462
Stacking (LR + KNN)	0.682692	0.682963	0.682692	0.682575	0.317308

A system of stacking configurations were explored systematically, including the combination of linear, distance-based, and tree-based models. The base learners were chosen by their good individual performance after selection of features whereas the meta-learner was trained on the individual predicted probabilities made by the base classifiers.

In all the combinations that were tested, it was found that the AdaBoost and K-Nearest Neighbors (AdaBoost + KNN) stacking model outperformed the other models in terms of overall performance. This architecture successfully merged the strong feature discrimination with KNN local neighborhood sensitivity (41). These models were complementary and thus allowed the ensemble to extrapolate effectively and reproduce the finer details of the data.

AdaBoost + KNN stacking ensemble had Accuracy: 0.7644, Precision: 0.7700, Recall: 0.7644, F1-score: 0.7631, Error Rate: 0.2355.

A total of 15 stacking ensemble configurations were tested to check if they can have better predictive performance when they use the two algorithms together. The common meta-learner applied was Logistic Regression, and the base learners were the combinations of Random Forest, Logistic Regression, K-Nearest Neighbors, Linear Support Vector Machine and AdaBoost. Five-fold cross-validation was used to generate out-of-fold predictions for creating the ensemble during the construction of the ensemble. The test set was an independent set, and was only used for last benchmarking purposes.

Among the evaluated stacking configurations, the comparative analysis demonstrated that combining heterogeneous classifiers can enhance predictive robustness compared with individual machine learning models. The systematic evaluation of multiple ensemble architectures enabled identification of the most effective classifier combination while preserving a leakage-aware evaluation protocol.

The stacking evaluation conducted using the leakage-free selected features reveals a different performance pattern compared to earlier experiments. Among all stacking configurations, as shown in **Figure 7**, Stacking (KNN + AdaBoost) achieved the highest accuracy (0.7644), observed accuracy among the evaluated stacking configurations. This finding suggests that the meta-model can benefit from a combination of the boosting-based learner and the distance-based classifier, which capitalizes on their complementary strengths. AdaBoost does a good job of adding nonlinear decision boundaries via iterative error correction, while KNN captures local neighborhood patterns. The combination of these structurally different learning mechanisms seems to give an advantage for predictive balance as indicated by the increased precision (0.7700) and F1-score (0.7632). Interestingly, this setup outperforms all stand-alone classifiers that are tested after feature selection, and thus demonstrates the value of meta-learning in this leakage-free environment.

**Figure 7:**
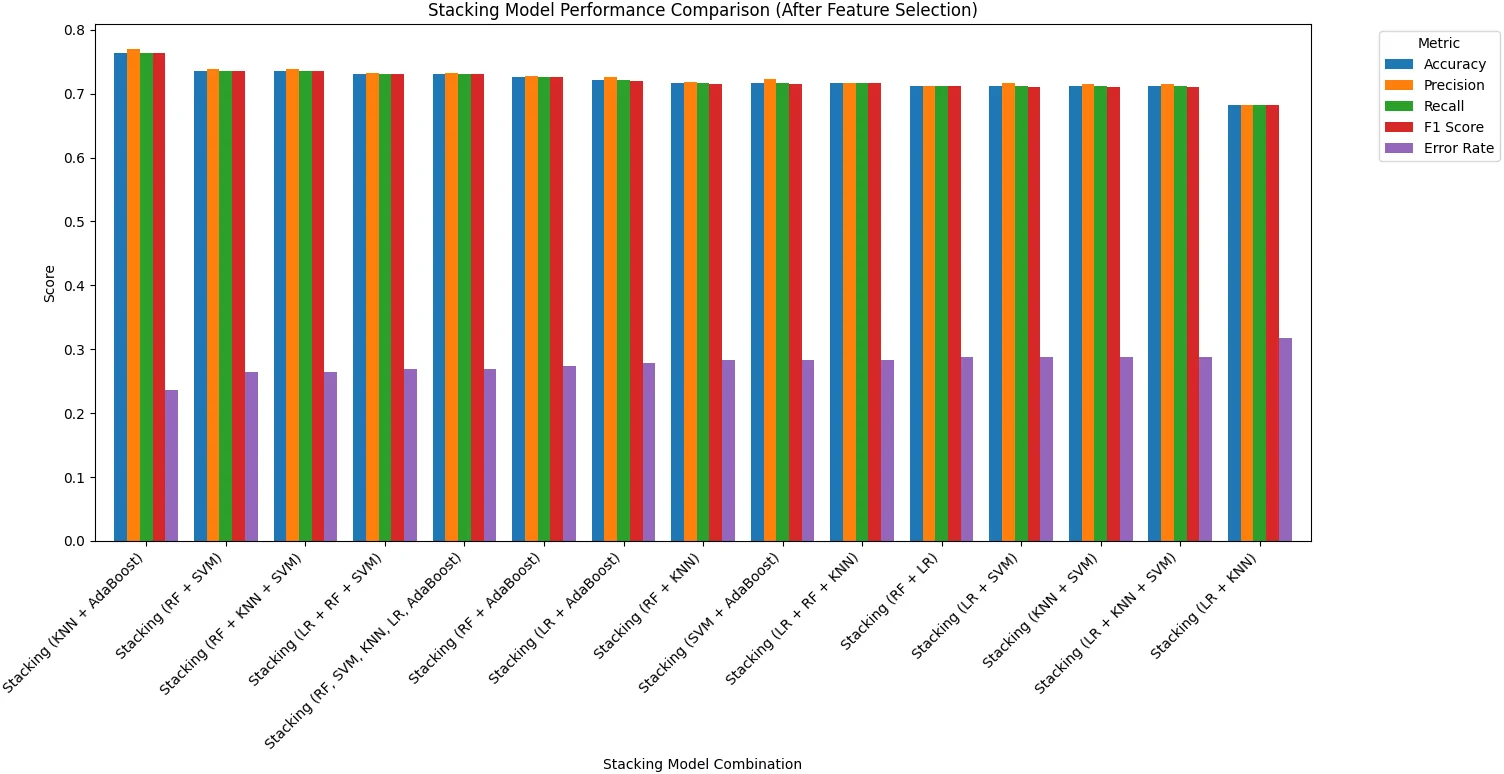
Stacking performance comparison.

The other stacking combinations (RF + SVM, RF + KNN+ SVM, LR + RF + SVM and five-model ensemble (RF, SVM, KNN, LR, AdaBoost)) achieved stable accuracy between 0.730- 0.736, which were slightly lower. Moderate improvement was achieved with such architectures as RF + AdaBoost, LR + AdaBoost and RF + KNN but these were not able to outperform KNN + AdaBoost pairing. The high dependency on linear learners (e.g., LR + SVM, LR + KNN) resulted in comparatively lower accuracy for combinations which indicated that there was in fact limited complementary diversity for the similar types of learners. Overall, the results of the stacking analysis are encouraging that meta-learning can make measurable gains over the single models; however, this is strongly dependent on both the diversity and interaction of the base learners. This leakage-free framework found the combination of AdaBoost and KNN as the most powerful approach for predicting the risk of ovarian cancer.

#### 4.5.1 Clinical performance evaluation

The balanced analytical cohort minimized the skewing effects from class imbalance when building models, but the overall accuracy may not be the most appropriate measure of diagnostic performance in the clinical setting. Thus, the stacking ensemble that performed best was subjected to additional analysis with clinically meaningful metrics used in diagnostic prediction studies. The measures offer complementary information about the model’s correct identification of cases of ovarian cancer while reducing false-positive and false-negative classifications. Confusion matrix derived clinical performance measures (Sensitivity, Specificity, Positive Predictive Value (PPV), Negative Predictive Value (NPV), Balanced Accuracy, Matthews Correlation Coefficient (MCC), ROC–AUC and PR–AUC) are summarized in **Table 8a**. These metrics reveal the strength and utility of the proposed leakage-aware prediction framework. The class-wise performance of the evaluated stacking models is presented in **Table 8B**.

**Table 8A:** Measurement of clinical performance for the top performing stacking ensemble using diagnostic metrics derived from a confusion matrix.

Model	Accuracy	Sensitivity	Specificity	PPV	NPV	Balanced accuracy	MCC	PR-AUC
Stacking (KNN + AdaBoost)	0.7644	0.6923	0.8365	0.8089	0.7310	0.7644	0.5344	0.8767
Stacking (RF + SVM)	0.7403	0.6826	0.7980	0.7717	0.7155	0.7403	0.4840	0.7948
Stacking (RF + KNN + SVM)	0.7403	0.6826	0.7980	0.7717	0.7155	0.7403	0.4840	0.7982
Stacking (RF + KNN)	0.7355	0.7211	0.7500	0.7425	0.7289	0.7355	0.4713	0.8582
Stacking (LR + RF + SVM)	0.7307	0.6826	0.7788	0.7553	0.7105	0.7307	0.4636	0.7979
Stacking (RF + AdaBoost)	0.7259	0.6923	0.7596	0.7422	0.7117	0.7259	0.4529	0.8640
Stacking (RF + LR)	0.7211	0.6923	0.7500	0.7346	0.7090	0.7211	0.4430	0.8510
Stacking (LR + AdaBoost)	0.7211	0.6442	0.7980	0.7613	0.6916	0.7211	0.4476	0.8350
Stacking (SVM + AdaBoost)	0.7163	0.6346	0.7980	0.7586	0.6859	0.7163	0.4385	0.7809
Stacking (LR + SVM)	0.7115	0.6346	0.7884	0.7500	0.6833	0.7115	0.4281	0.7656
Stacking (LR + KNN + SVM)	0.7115	0.6442	0.7788	0.7444	0.6864	0.7115	0.4269	0.7719
Stacking (KNN + SVM)	0.7115	0.6442	0.7788	0.7444	0.6864	0.7115	0.4269	0.7713
Stacking (LR + RF + KNN)	0.7115	0.6826	0.7403	0.7244	0.7000	0.7115	0.4237	0.8489
Stacking (LR + KNN)	0.6971	0.6730	0.7211	0.7070	0.6880	0.6971	0.3946	0.7306

**Table 8B:** Class-wise performance of the best model.

Model	Precision (Class 0)	Recall (Class 0)	F1 (Class 0)	Precision (Class 1)	Recall (Class 1)	F1 (Class 1)
Stacking (KNN + AdaBoost)	0.7310	0.8365	0.7802	0.8089	0.6923	0.7461
Stacking (RF + SVM)	0.7155	0.7980	0.7545	0.7717	0.6826	0.7244
Stacking (RF + KNN + SVM)	0.7155	0.7980	0.7545	0.7717	0.6826	0.7244
Stacking (RF + KNN)	0.7289	0.7500	0.7393	0.7425	0.7211	0.7317
Stacking (LR + RF + SVM)	0.7105	0.7788	0.7431	0.7553	0.6826	0.7171
Stacking (RF + AdaBoost)	0.7117	0.7596	0.7348	0.7422	0.6923	0.7164
Stacking (RF + LR)	0.7090	0.7500	0.7289	0.7346	0.6923	0.7128
Stacking (LR + AdaBoost)	0.6916	0.7980	0.7410	0.7613	0.6442	0.6979
Stacking (SVM + AdaBoost)	0.6859	0.7980	0.7377	0.7586	0.6346	0.6910
Stacking (LR + SVM)	0.6833	0.7884	0.7321	0.7500	0.6346	0.6875
Stacking (LR + KNN + SVM)	0.6864	0.7788	0.7297	0.7444	0.6442	0.6907
Stacking (KNN + SVM)	0.6864	0.7788	0.7297	0.7444	0.6442	0.6907
Stacking (LR + RF + KNN)	0.7000	0.7403	0.7196	0.7244	0.6826	0.7029
Stacking (LR + KNN)	0.6880	0.7211	0.7042	0.7070	0.6730	0.6896

Comparative performance tables show weighted-average Precision, Recall, and F1-score while class-specific figures for the top model are shown separately for a clinically more relevant comparison.

The proposed stacking framework demonstrated good diagnostic performance for both classes, which indicated that the framework could correctly identify ovarian cancer cases with a low false positive rate. The high balanced accuracy and MCC also indicate that the proposed framework not only provides reliable discrimination but also is meaningful beyond just overall accuracy. Beyond that, the PR–AUC is found to be effective in discriminating the positive ovarian cancer cases and is especially significant in screening applications where the prevalence of the disease is relatively smaller.

#### 4.5.2 Statistical comparison of model performance

To estimate the uncertainty of the performance estimates reported, 95% bootstrap confidence intervals were calculated with respect to the best-performing models. Moreover, the predictions of the proposed KNN–AdaBoost stacking ensemble model were compared with that of the best-performing individual classifier (AdaBoost) using McNemar’s test. The McNemar’s test gave a statistic of 0.45 and a p value of 0.5023 which are not significant because the difference in predictive capacities is not statistically significant. The stacking ensemble, therefore, should not be taken to be a statistically better ensemble than AdaBoost; its performance should be compared to that of AdaBoost. The corresponding 95% confidence intervals are presented in **Table 9**. The statistical comparison between AdaBoost and the KNN–AdaBoost stacking model is presented in **Table 10**.

**Table 9:** Confidence interval.

Ranking	Model	Accuracy	95% CI lower	95% CI upper
9	Stacking (KNN + AdaBoost)	0.764423	0.701923	0.817428
10	Stacking (RF + KNN + SVM)	0.740385	0.682692	0.793269
1	Stacking (RF + SVM)	0.740385	0.677885	0.798077
2	Stacking (RF + KNN)	0.735577	0.673077	0.793269
14	Stacking (RF, SVM, KNN, LR, AdaBoost)	0.735577	0.673077	0.793269
12	Stacking (LR + RF + SVM)	0.730769	0.668269	0.788462
3	Stacking (RF + AdaBoost)	0.725962	0.663462	0.783774
6	Stacking (LR + AdaBoost)	0.721154	0.663341	0.778846
0	Stacking (RF + LR)	0.721154	0.658654	0.783654
8	Stacking (SVM + AdaBoost)	0.716346	0.653726	0.778846
5	Stacking (LR + SVM)	0.711538	0.649038	0.774159
11	Stacking (LR + KNN + SVM)	0.711538	0.649038	0.774038
7	Stacking (KNN + SVM)	0.711538	0.649038	0.774038
13	Stacking (LR + RF + KNN)	0.711538	0.648918	0.769351
4	Stacking (LR + KNN)	0.697115	0.634615	0.759615

**Table 10:** Statistical comparison between AdaBoost and the proposed KNN–AdaBoost stacking model.

Comparison	Test	Statistic	p-value	Interpretation
AdaBoost vs. KNN–AdaBoost Stacking	McNemar’s Test	0.45	0.5023	No statistically significant difference

### 4.6 Calibration and clinical utility analysis

In order to evaluate the reliability and feasibility of the proposed model, calibration and decision-curve analyses were conducted with respect to KNN–AdaBoost stacking ensemble. The model performance with a Brier score of 0.1686 showed the overall probabilistic prediction accuracy of the model in a good level. The calibration intercept was 0.0021, close to the desired value of 0, suggesting very little systematic bias in the predicted probabilities. But the calibration slope was 2.4486, which is away from one, and shows that the risk level was higher than the predicted level. Further recalibration of the model may be needed before its probabilities are applied directly to clinical risk estimation, however, due to the overall good probability accuracy of the model. The calibration curve is displayed in **Figure 8**, indicating the consistency between the predicted and observed probabilities of events.

**Figure 8:**
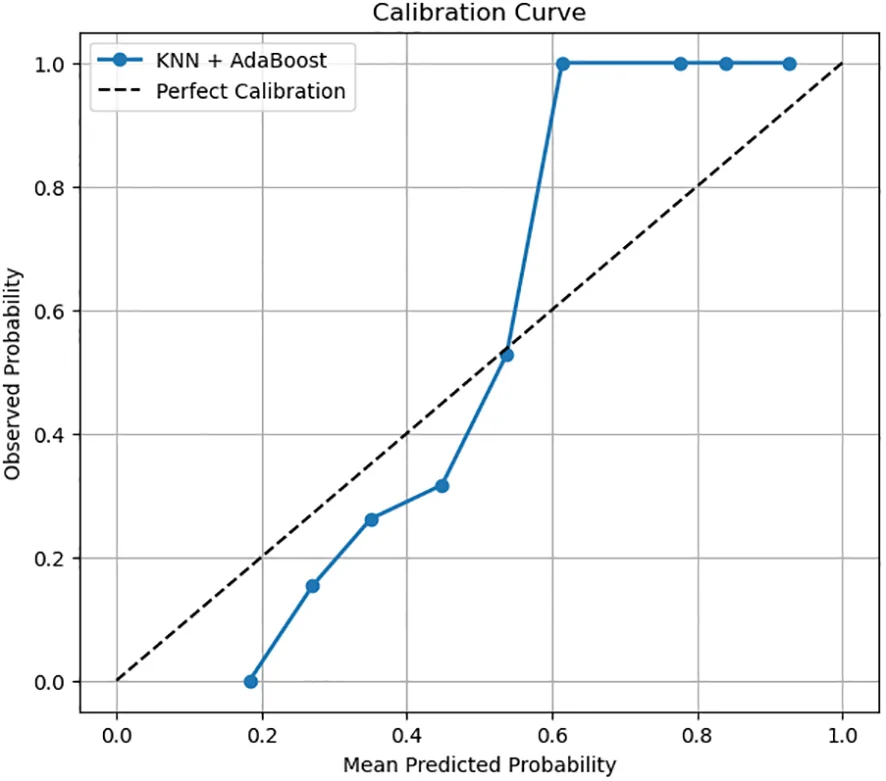
Calibration curve of the KNN–AdaBoost stacking model.

A decision curve analysis was then conducted using prevalence adjusted predicted probabilities because there was a significant difference between the prevalences of the analytical cohort and the original PLCO ovarian cancer cohort. For various values of the threshold probability, the net benefit of the KNN-AdaBoost model was compared to the treat-all and treat-none strategies. This decision curve is shown in **Figure 9**. The analysis was exploratory in nature, since the model was built on an undersampled analytical cohort and has yet to be externally validated on a cohort that is representative of the general population.

**Figure 9:**
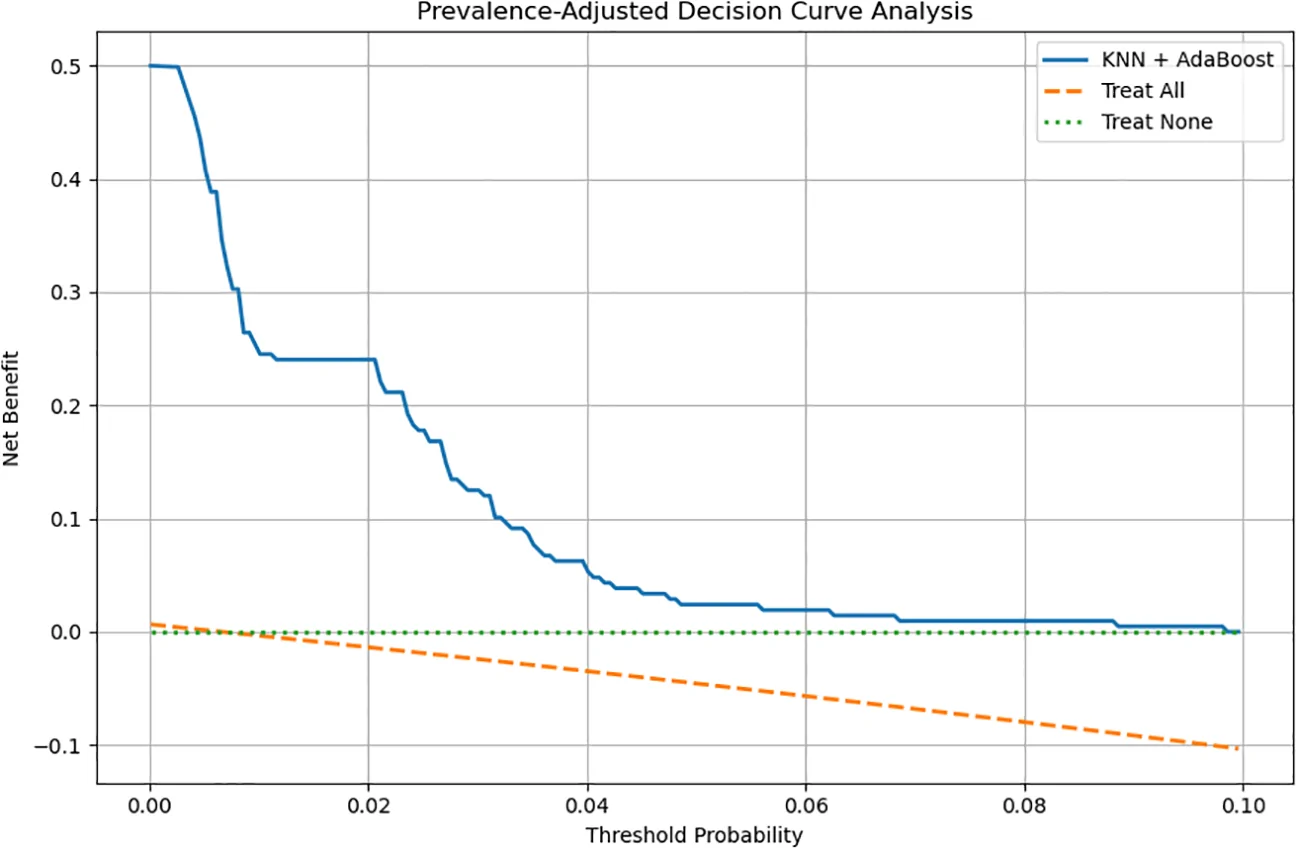
Prevalence-adjusted decision curve analysis.

### 4.7 Explainability analysis (XAI).

A thorough Explainable Artificial Intelligence (XAI) analysis was done to make sure that the proposed predictive model is not only an accurate one but also a clinically interpretable one. Since ensemble models are complex and the problem of transparency in medical decision-support systems is ethically important, model interpretability was measured by SHAP (SHapley Additive Explanations) and Permutation Importance (PI). The approaches give information pertaining to the role of individual features in making predictions based on the model, so that clinicians and scientists can determine the logic behind the risk estimates.

#### 4.7.1 SHAP summary insights

The full KNN–AdaBoost stacking model was interpreted using model-agnostic SHAP analysis. A representative subset of 50 observations was sampled randomly from the training set, and for 200 observations sampled randomly from the independent test set, SHAP explanations were produced. The entire stacking predictor function (both the base learners and meta-classifier) was fed to the SHAP explainer, and feature-attribution values were computed for the overall stacking prediction without averaging over the single base learners.

##### 4.7.1.1 Top SHAP-contributing predictors

The SHAP summary plots showed that the strongest predictor of the updated model is as illustrated in the **Figure 10** SHAP Summary Plot, which comprise:

- Ph_ovar_sqx
- Ph_ovar_dhq
- Entryage_sqx
- Ca125_result5
- Entrydays_sqx
- Age
- tvu_result0

**Figure 10:**
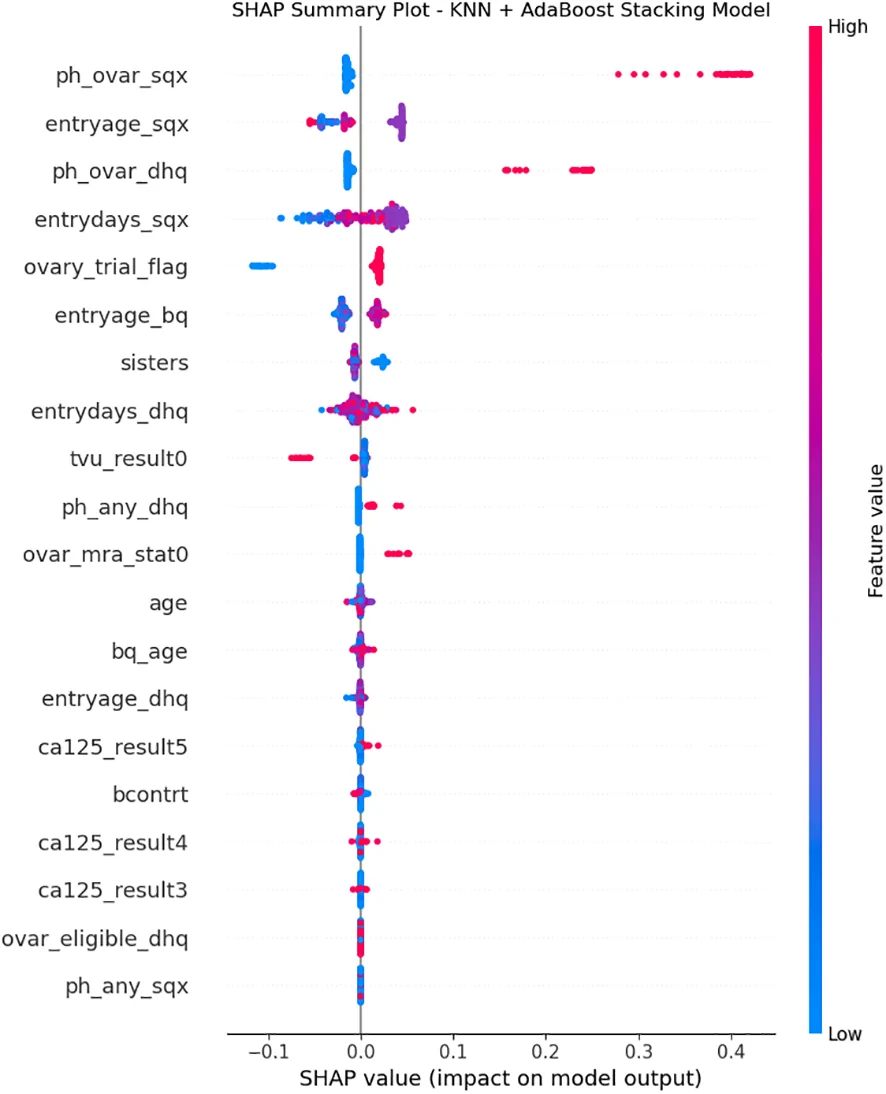
SHAP summary plot.

These features had the highest absolute SHAP values, which shows that they have a significant influence on the outcome of predicted ovarian cancer amongst the patients. Notably, no mortality-related or post-diagnostic variables emerged in the list of the dominant predictors, which proves that the model is based on the clinically available information that is pre-diagnostic.

##### 4.7.1.2 Interpretation of feature effects

The variable ph_ovar_sqx proved to be the most powerful predictor, which indicates that the responses to questionnaire-based ovarian screening can signal a lot. This emphasizes the significance of patient-reported gynecological history and screening-related items in the detection of risks at an early stage. Likewise, ph_ovar_dhq is another study that intensifies the usefulness of systematized health questionnaires in eliciting pre-diagnostic symptoms or risk factors.

The age variables, such as entryage_sqx and age, were also found to cause consistent contributions, which is in line with the existing epidemiological findings that ovarian cancer risk rises with age. Entrydays_sqx suggests that the engagement indicators related to health might be in terms of patterns of engagement and gaps in engagement.

Other features of Biomarker and Imaging, such as a new ca125 result5, and the TVU result0, all demonstrated clinical value in the diagnosis of ovarian cancer that was established clinically, played an important role. The model has appropriate validated clinical items and has high CA-125 and ultrasound levels, which are well-known screening items, and are endorsed by contributions from the SHAP.

##### 4.7.1.3 Clinical implications of SHAP findings

The SHAP analysis illustrates that the updated, leakage-free model goes on to make predictions on the basis of the consistent merge of questionnaire-collected information, age-associated indicators, screening attendance measurements, and biomarker outcomes. This is a multidimensional but clinically interpretable framework that increases the credibility and makes its possible translation to clinical use. SHAP allows clinicians to explain the risk independently on a patient-level, making it easy to comprehend the factors influencing each decision, and furthermore, it can be applied to make informed clinical decisions in the future and improve the implementation of the system in ovarian cancer risk stratification practices.

### 4.8 Permutation importance

The permutation importance (PI) was calculated to test the performance decline of the model when the values of each feature were randomly permuted, and hence the relationship between the features and the outcome variable was interrupted. The method offers a strong, model-free approximation of the extent to which each predictor would remain important in upholding the overall accuracy of the classifier. Permutation importance uses the loss in performance incurred after reshuffling a feature in the training model to determine the actual predictive value of that variable in the model.

Two versions of PI were calculated:

1. Standard permutation importance, which is computed based on the single fitted model.
2. Cross-validated permutation importance averages importance scores as across many folds to decrease variance and limit reliance on a particular train test split.

#### 4.8.1 Top predictors identified through permutation importance

The highest-scoring features, based on their contribution to predictive accuracy in the leakage-free model, were:

- ph_ovar_sqx (Importance: 0.121)
- entryage_sqx (Importance: 0.023)
- entrydays_sqx (Importance: 0.020)
- entrydays_dhq (Importance: 0.019)
- ph_ovar_dhq (Importance: 0.014)

**Top 20 Features by PI**

**Feature Importance Std**

ph_ovar_sqx 1.206731e-01 0.018174

entryage_sqx 2.307692e-02 0.014865

entrydays_sqx 1.971154e-02 0.017261

entrydays_dhq 1.923077e-02 0.009122

ph_ovar_dhq 1.394231e-02 0.011468

ca125_result3 8.653846e-03 0.006731

age 5.288462e-03 0.007586

tvu_result0 4.326923e-03 0.006250

ca125_result5 3.846154e-03 0.012314

ca125_result4 3.365385e-03 0.004327

entryage_dhq 4.440892e-17 0.008600

ph_any_dhq -1.442308e-03 0.013776

ovary_trial_flag -1.442308e-03 0.006468

ovar_eligible_dhq -1.442308e-03 0.003078

entryage_bq -2.403846e-03 0.008667

bq_age -3.846154e-03 0.004711

ph_any_sqx -3.846154e-03 0.004711

ovar_mra_stat0 -5.288462e-03 0.008173

sisters -8.653846e-03 0.015020

bcontrt -9.134615e-03 0.014335

**Built-in Feature Importance (Top 20)**

**Feature Importance**

ph_ovar_sqx 0.320203

ph_ovar_dhq 0.084182

entryage_sqx 0.081163

ovary_trial_flag 0.073954

ph_any_dhq 0.055398

ca125_result5 0.050737

entrydays_sqx 0.039030

ca125_result3 0.029266

ovar_mra_stat0 0.028652

entryage_dhq 0.026517

bcontrt 0.025703

ph_any_sqx 0.024377

sisters 0.023867

entryage_bq 0.022922

entrydays_dhq 0.022571

tvu_result0 0.020885

age 0.020044

bq_age 0.018389

ovar_eligible_dhq 0.018332

ca125_result4 0.013808

Additional meaningful contributors included ca125_result3 (0.0087), age (0.0053), and tvu_result0 (0.0043). Such variables generated the highest declines in model performance in permutation, which means that they are crucial in maintaining ovarian cancer prediction accuracy. It is worth mentioning that, according to **Figure 11**, Permutation Importance and Built-in Feature Importance, ph_ovar_sqx had a significantly higher importance score than any other feature, which shows its domineering powers in the model.

**Figure 11:**
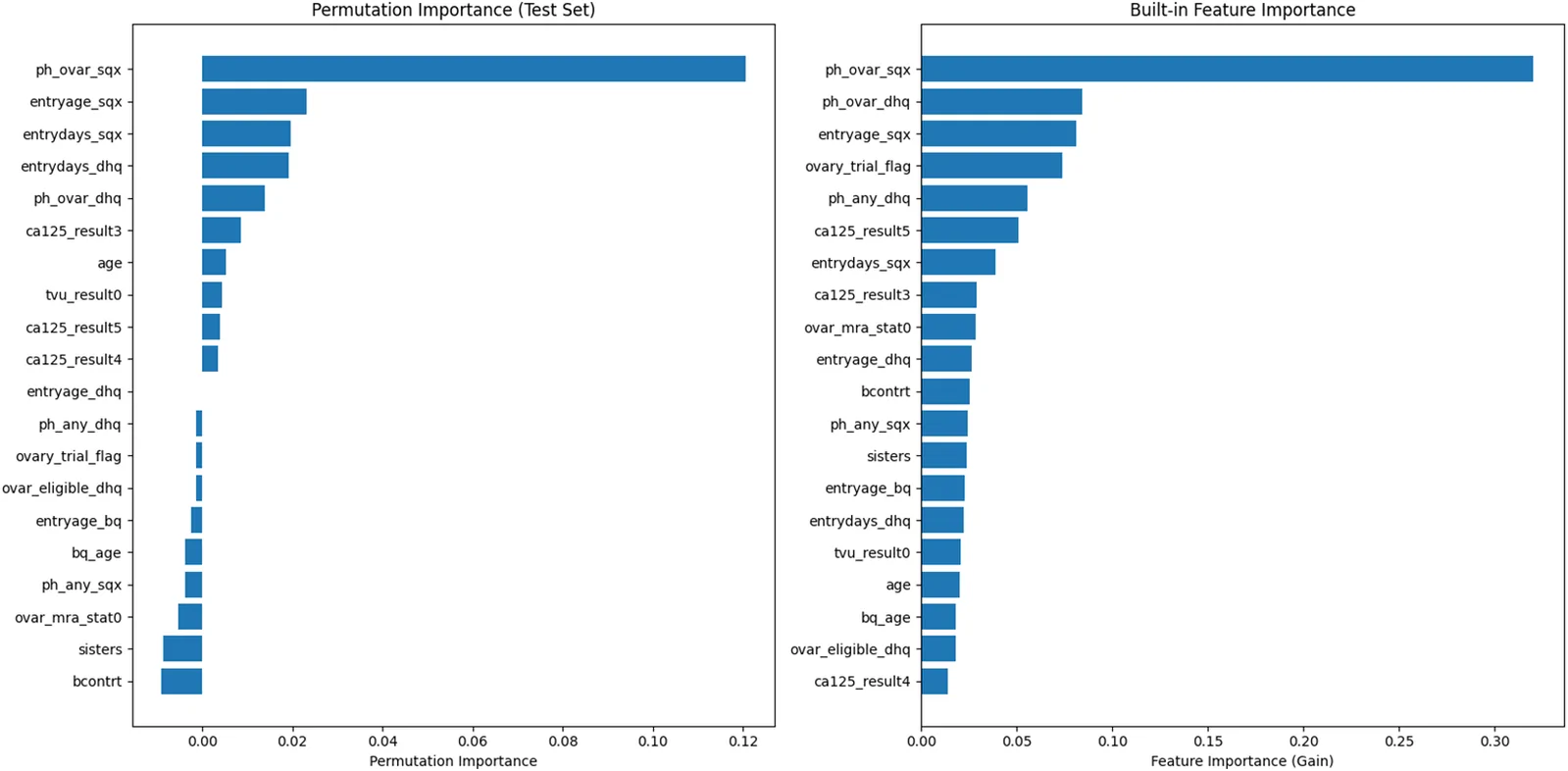
Permutation importance and built-in feature importance.

#### 4.8.2 Interpretation of PI findings

The results of the permutation show that the model mainly depends on the responses of the screening questionnaire, age-at-entry factors, screening timing factors, and CA-125 biomarker level additions to come up with the predictions. The high significance of ph_ovar_sqx emphasizes the predictive ability of screening information provided by participants and gynecological health reactions. On the same note, there are entry-age variables (entryage_sqx) and screening timing characteristics (entrydays_sqx, entrydays_dhq) which are indicative of the applicability of age-related and screening participation trends in risk estimation. Biomarker variables, including ca125 result3 and imaging variables, including tvu result 0, also confirm the roles of the known clinical screening variables.

#### 4.8.3 Consistency across interpretability methods

The key strength of the updated review is that it arrives at a shared outcome between a series of independent interpretability systems, such as:

- SHAP (SHapley Additive Explanations)
- Permutation importance
- Intrinsic model feature significance.

In all these methods, there was a series of similar predictors that became potent contributors to predicting risks of ovarian cancer. Specifically, phovar sqx was singled out as the most significant feature on multiple occasions, being the most important during permutation analysis and the magnitude of SHAP. Other predictors that were always brought to the fore were ph_ovar_dhq, entryage_sqx, entrydays_sqx, ca125_result5, and age. Both filter-based selection and model-based importance and *post hoc* explanation methods have shown consistency in feature relevance. Notably, none of the variables related to mortality or post-diagnostic was among the leading predictors, which validates the methodological rigor of the leakage-free framework of modeling.

This multi-method alignment is a great contribution to the believability of the predictive model. Independent analytical strategies that are similar in the features that they target reduce the risk that the order of importance is because of a certain algorithm, sampling variation, or modeling error. Instead the results show that the model reflects informative and reproducible risk indicators truly.

#### 4.8.4 Clinical and methodological implications

The results offer internal validation of the predictive capacity and interpretability of the proposed framework but the external, temporal and prospective validation is needed before clinical implementation. All of the screening variables of the questionnaire (ph ovar sqx, ph ovar dhq), age variables (entryage sqx, age), screening timing variables (entrydays sqx) and ovarian cancer biomarker outcomes (ca125 result5) are appropriate and consistent with the known epidemiological and screening-based risk factors for OVC. This will make the model more biologically plausible and more translationally potential to real-world risk stratification.

Technically, the findings of the overlap of SHAP, permutation importance, and internal model importance suggest a way to emphasize the importance of using complementary interpretability tools in medical AI research. This redundancy ensures strength, reproducibility, and transparency, crucial aspects in the ethical implementation of AI in healthcare settings. The model has all the essential properties of a reliable AI model, which are explainability, validity, and fairness, because clinically meaningful pre-diagnostic factors drive its predictive performance, suggesting it has potential to contribute to the screening processes of ovarian cancer.

### 4.9 Summary of findings

The study overall showed the feasibility of a leakage-aware machine-learning approach for ovarian cancer risk prediction using the PLCO data that is both interpretable and leakage-aware. The four-method feature-selection strategy was able to select 20 features and give a compact and easy-to-explain feature set, and with the KNN–AdaBoost, the highest observed value of stacking accuracy (0.7644) was obtained. This performance was not statistically different from AdaBoost (McNemar test: statistic = 0.45, p = 0.5023), however. The screening-related variables, age, and CA-125 were consistent influential predictors as determined by SHAP and permutation analysis. The additional analyses of the diagnostic, calibration and clinical-utility provided a wider assessment of model performance. The findings should be viewed as internal evaluation and exploratory, and should be confirmed externally, temporally and prospectively before clinical use.

## 5 Discussion

From the current research, it is shown that the model for prediction of ovarian cancer based on PLCO screening data is a strictly-structured machine learning model which is leakage sensitive. The architecture will have a balance between predictive and clinical interpretability by embedding structured preprocessing, multi-method feature selection, ensemble modeling, and elaborate explainability analysis. The results show that the well-designed ensemble model is able to carry out good discrimination, is transparent, and has a good method. This section puts the findings into perspective as they relate to the available literature, presents implications of the methodologies, and presents future research and clinical translation directions.

### 5.1 Principal findings

The main finding of this work is the discovery of a highly performing stacking ensemble of KNN + AdaBoost which has the best overall accuracy (0.7644) of all the tested models under the leakage-free condition. Although when compared individually, it was shown that boosting algorithms like XGBoost, CatBoost, and LightGBM performed well (with AdaBoost showing the highest single classifier accuracy (0.7452)) stacking configuration offered a quantifiable boost to the performance, by synergizing complementary learning behavior. AdaBoost brings healthy nonlinear decision limits and solid structured tabular data manipulation, and KNN, local prevalent patterns in the feature space. The meta-learning fusion minimized the variation and improved predictive equilibrium in charges of precision, recall and F1-score.

The other one is the ‘multi-method’ feature selection strategy. ANOVA, Chi-square testing, and both the tree-based feature importance and ranking were used to reduce the original high dimensional data set to 20 clinically relevant and pre-diagnostic predictors. This dimensionality reduction enhanced computational efficiency as well as minimized overfitting and retained relevant predictive signals. Interestingly, the latter set of features failed to take mortality and post-diagnostic factors into account, and hence did not reflect retrospective artifacts, but rather realistic pre-detection risk estimation.

A key finding was that feature selection had no effect on prediction accuracy on its own. The best stand-alone AdaBoost model achieved 0.7644 accuracy with the original features, while with the selected features it achieved 0.7452 accuracy. But when KNN and AdaBoost were stacked after feature selection, the accuracy was brought back to 0.7644. Therefore, the proposed framework should not be viewed as adding to the accuracy boost due to only feature selection. Instead, the feature selection approach resulted in a smaller representation of features, and the stacking scheme ultimately restored predictive power over these smaller dimensions.

### 5.2 Comparison with prior studies

Earlier research on predicting the occurrence of ovarian cancer has mainly been based on classical statistical models or univariate analysis of biomarkers such as CA-125 and transvaginal ultrasound (4, 5). Even though recent work has used machine learning methods, many models are either characterized by low interpretability or a small sample size. The results of our study add to the body of literature because they show that by combining various machine learning techniques with explainability technologies, it is possible to achieve both predictive and clinically understandable results.

The current research contributes to this body of work in a number of aspects. To begin with, it uses a high population-based cohort data with multi-domain variables, such as demographic, screening participation indicators, biomarker findings, and questionnaire-based indicators. This multi dimensional method allows the model to take into consideration the minor interactions which could be missed by biomarker based models. Second, this framework combines SHAP analysis, permutation importance, and cross-method validation to provide transparency, unlike most previous ML research which focuses on predictive accuracy, not decipherable.

Though in the leakage-free environment, predictive accuracy is more conservative than in the previously conducted experimental experiments, the findings give a methodologically sound and clinically credible estimate of actual predictive ability. Such rigor increases the reproducibility of the findings and increases the scientific validity.

### 5.3 Clinical and methodological implications

The present study demonstrates that integrating leakage-aware preprocessing, consensus-based feature selection, ensemble learning, and explainable artificial intelligence can produce an interpretable framework for ovarian cancer risk prediction using population-based screening data. A The key methodological advantage of the proposed framework is the systematic analysis of several stacking ensemble architectures. Instead of using a single ensemble setup, multiple setups with various combinations of different heterogeneous classifiers have been tested with Logistic Regression as the common meta-learner. Five-fold cross-validation was used to obtain out-of-fold predictions for the training of the meta-models, helping to avoid overfitting and to prevent information leakage across the learning stages. This ensemble approach enhances the strength and reliability of the proposed framework and offers a thorough evaluation of different combinations of classifiers.

The study has certain methodological advantages:

1. All missing values, categorical variables, and outliers were cleaned up in the preprocessing pipeline to achieve consistency.
2. Data leakage variables were explicitly removed, and temporal and clinical validity was maintained.
3. Using a multi-method feature selection technique reduced the number of redundant features and increased the generalizability of the models.
4. Benchmarking of classical models, ensemble models, and stacking models enabled comparisons.
5. We used SHAP, permutation importance, and cross-validated importance scores to guarantee interpretability and stability.

These methodological choices create a more consistent and consistent prediction model as a consequence. In addition to discrimination, calibration and clinical utility were also assessed to give a more complete picture of the proposed framework. The Brier score (0.1686) and calibration intercept (0.0021) were satisfactory in terms of the overall probability accuracy and systematic bias in the predictions, respectively. A calibration slope of 2.4486, however, showed deviation from ideal calibration, meaning that the predicted probabilities were possibly not absolute individual risks and may need to be recalibrated before they can be interpreted. However, the prevalence adjusted DCA was an exploratory measure to see if there was potential clinical benefit, but due to random undersampling the underpinning analytical cohort should not be viewed as definitive evidence of clinical benefit. Before applying to personalized screening or clinical decision-support systems, external validation with a population-representative cohort and prospective evaluation is needed.

In recent years, there have been several oncology studies that underscore the role of explainable AI in clinically relevant cancer prediction and diagnosis tasks, such as breast cancer risk stratification (42), leukemia diagnosis (43), and brain tumor detection (44). The results validate the use of interpretable AI, in addition to predictive performance. In the current study, SHAP and permutation-based analyses offer insight into the features driving an ovarian cancer risk prediction in a leakage-aware framework.

### 5.4 Limitations of the present study

Several limitations should be acknowledged. First, random undersampling was used to address the severe class imbalance, which reduced the control population and may not reflect the original disease prevalence. Second, although post-diagnostic variables were excluded, the PLCO dataset contains longitudinal screening information; therefore, the framework should be considered a pre-diagnostic risk-stratification model rather than a baseline-only model with a fixed prediction horizon. Third, evaluation was performed within a single PLCO cohort without temporal, external, or prospective validation. Although calibration and decision-curve analyses were conducted, their findings require confirmation in population-representative external cohorts. The KNN–AdaBoost ensemble also did not show a statistically significant improvement over AdaBoost (McNemar statistic = 0.45, p = 0.5023), indicating comparable rather than superior performance. Finally, the reported diagnostic, calibration, and clinical-utility measures should be considered exploratory, and external multicenter and prospective validation, subgroup assessment, and recalibration are required before clinical implementation.

### 5.7 Implications for future screening approaches

The proposed framework demonstrates the potential of integrating demographic, questionnaire-derived, screening, and biomarker information for ovarian cancer risk stratification. The additional calibration and decision-curve analyses provide an initial assessment of probability reliability and potential clinical utility. However, the observed calibration slope (2.4486) indicates that further recalibration may be required before the predicted probabilities can be interpreted as absolute individual risks. Similarly, the decision-curve analysis should be considered exploratory because the model was developed using an undersampled analytical cohort.

Future studies should therefore focus on external validation in population-representative cohorts, probability recalibration, prospective clinical evaluation, and assessment of clinical utility before implementation in personalized screening or clinical decision-support workflows. Further investigation of fairness, longitudinal biomarker trajectories, and multimodal information, including imaging features, may also determine whether these additions improve predictive performance and clinical usefulness.

## 6 Conclusion

In this paper, we propose and test a leakage-aware, interpretable machine learning model to predict ovarian cancer using the PLCO screening dataset. A complete set of clinically relevant preprocessing predictors was identified by using a multi-method feature selection strategy, while rigorous preprocessing ensured efficient dimensionality reduction without losing the predictive integrity of the pre-diagnostic information. The leakage-aware KNN–AdaBoost stacking framework proposed in this paper exhibited competitive predictive performance for ovarian cancer prediction. The proposed ensemble architecture was verified as effective without making any unsupported claims of its statistical superiority because the results of a statistical comparison of its predictive accuracy are similar to the best individual classifier (AdaBoost). The stacking ensemble of KNN and AdaBoost was found to be the most effective configuration, highlighting the benefit of combining complementary learning paradigms for boosting performance in terms of generalization. Importantly, the interpretation analyses (SHAP, permutation importance) indicated the same variables from the screening questionnaire, age-related indicators, and CA-125 features as being the most important variables, reinforcing the clinical plausibility of the model. This underscores the need for methodologic rigor and transparency and offers a reproducible and clinically relevant approach to ovarian cancer risk stratification. A key limitation of this study is the absence of external validation. Future work should include external validation, longitudinal modeling, and multimodal integration to be translated into real-world screening applications.

## Data Availability

The data analyzed in this study is subject to the following licenses/restrictions: The PLCO dataset is not publicly available because access is restricted by the National Cancer Institute (NCI) Cancer Data Access System (CDAS) data-use requirements. Qualified researchers may request access through CDAS, subject to NCI approval and the applicable data-use agreement. The authors are not permitted to redistribute or publicly share the dataset. Requests to access these datasets should be directed to PLCO Data Access – NCI Cancer Data Access System (CDAS): https://cdas.cancer.gov/plco/. The data used in this study were obtained from the Prostate, Lung, Colorectal, and Ovarian (PLCO) Cancer Screening Trial through the National Cancer Institute (NCI) Cancer Data Access System (CDAS) under approved project PLCOI-1591, entitled “Innovative approaches towards early stage ovarian cancer prediction using Artificial Intelligence.” The data were accessed in accordance with the applicable CDAS data-use requirements.
