# Supplementary material for: A leakage-aware and interpretable machine learning framework for ovarian cancer prediction using the PLCO screening dataset
Source: Front Oncol. 2026 Oct 30;16:1926361. doi: 10.3389/fonc.2026.1926361 (PMC13759362; doi:10.3389/fonc.2026.1926361)
Supplement: Supplementary file 1 [file Table1.docx]

**Supplementary Table S1. Model Hyperparameters and Configuration**

| **Model** | **Key hyperparameters** | **Random seed** |
| --- | --- | --- |
| Logistic Regression | max_iter=200 | 42 |
| Linear SVM | kernel=linear | 42 |
| KNN | n_neighbors=5 | — |
| Random Forest | n_estimators=100, max_depth=10, min_samples_split=20, min_samples_leaf=10, max_features=sqrt | 42 |
| Decision Tree | max_depth=5, min_samples_split=30, min_samples_leaf=15 | 42 |
| AdaBoost | n_estimators=100, learning_rate=1.0, base tree max_depth=1 | 42 |
| LightGBM | Exact parameters from notebook | 42 |
| CatBoost | Exact parameters from notebook | 42 |
| XGBoost | Exact parameters from notebook | 42 |
| KNN–AdaBoost Stacking | KNN + AdaBoost; Logistic Regression meta-classifier; cv=5 | 42 |

**Supplementary Table S2. Feature Selection and Model-Selection Procedure**

| **Component** | **Procedure** |
| --- | --- |
| Feature-selection methods | ANOVA, Chi-square, Random Forest importance, XGBoost importance |
| Rank aggregation | Sum of ranks |
| Final number of features | 20 |
| Data split | 80:20 |
| Stratification | Yes |
| Random seed | 42 |
| Class balancing | Random undersampling |
| Stacking CV | 5-fold |
| Final stacking model | KNN + AdaBoost |
| Meta-classifier | Logistic Regression |

**Supplementary Table S3. Computational Environment and Software Versions**

| **Component** | **Version** |
| --- | --- |
| Python | 3.13.5 |
| NumPy | 2.1.3 |
| Pandas | 2.2.3 |
| scikit-learn | 1.6.1 |
| SHAP | 0.52.0 |
| XGBoost | 3.0.4 |
| LightGBM | 4.6.0 |
| CatBoost | 1.2.8 |
| Environment | Anaconda, 64-bit |
